# Cytoreductive surgery plus hyperthermic intraperitoneal chemotherapy improves survival for peritoneal carcinomatosis from colorectal cancer: a systematic review and meta-analysis of current evidence

**DOI:** 10.18632/oncotarget.17497

**Published:** 2017-04-27

**Authors:** Chao-Qun Huang, Yao Min, Shu-Yi Wang, Xiao-Jun Yang, Yang Liu, Bin Xiong, Yutaka Yonemura, Yan Li

**Affiliations:** ^1^ Department of Gastrointestinal Surgery, Zhongnan Hospital of Wuhan University, Hubei Cancer Clinical Study Center & Hubei Key Laboratory of Tumor Biological Behaviors, Wuhan Clinical Research Center for Peritoneal Carcinomatosis, Wuhan, P.R. China; ^2^ Department of Ophthalmology, Central Hospital of Wuhan Affiliated to Tongji Medical College of Huazhong University of Science and Technology, Wuhan, P.R. China; ^3^ NPO to Support Peritoneal Surface Malignancy Treatment, Osaka, Japan; ^4^ Department of Peritoneal Cancer Surgery, Beijing Shijitan Hospital of the Capital Medical University, Beijing, P.R. China

**Keywords:** colorectal cancer, peritoneal carcinomatosis, cytoreductive surgery, hyperthermic intraperitoneal chemotherapy, meta-analysis

## Abstract

**Objectives:**

The therapeutic efficacy of cytoreductive surgery (CRS) plus hyperthermic intraperitoneal chemotherapy (HIPEC) for patients with peritoneal carcinomatosis (PC) from colorectal cancer (CRC) is still under debate. This meta-analysis and systematic review of published literature on this comprehensive strategy aims to evaluate its efficacy on CRC patients with PC.

**Methods:**

A systemic review with meta-analysis of published literatures on treatment of CRS plus HIPEC for patients with PC from CRC was performed. In addition, a summary of study results of published literatures concerning CRS plus HIPEC treating patients with PC from CRC was also conducted.

**Results:**

A total of 76 studies were selected, including 1 randomized controlled trial, 14 non-randomized controlled studies, and 61 non-controlled studies. The pooled hazard ratios (HRs) for overall survival (OS) in the 15 researches for meta-analysis was 2.67 (95% CI, 2.21-3.23, *I*_2_= 0%, *P* < 0.00001), and no significant evidence of publication bias was found. The difference of chemotherapy regimens of HIPEC was not associated with OS and DFS (disease-free survival) after CRS and HIPEC, with no significant difference of heterogeneity (*P* = 0.27, *I2* = 24.1%). In both groups of mitomycin C based HIPEC group and oxaliplatin group, patients received HIPEC had significant better survival (*P* < 0.00001). The mean mortality and morbidity for HIPEC program were 2.8% and 33.0%, respectively.

**Conclusions:**

This meta-analysis revealed that comprehensive therapeutic strategy of CRS plus HIPEC could bring survival benefit for selected patients with PC from CRC with acceptable safety.

## INTRODUCTION

Peritoneal carcinomatosis (PC), as a lethal regional progression for patients with colorectal cancer (CRC), has long been considered as a terminal condition with few effective treatments. In the past, the median overall survival (OS) of PC from colorectal cancer is 4 to 7 months after palliative surgery or 5-FU-based systemic chemotherapy with best supportive care [1-3]. Current systemic chemotherapy focusing on new chemotherapeutic agents such as oxaliplatin and irinotecan, along with anti-angiogenesis molecular targeting agents cetuximab and bevacizumab [4-7], could extend the median OS up to about 12 months [5]. However, long-term survival is still hard to be achieved by systemic chemotherapy alone.

Researches on treatment of CRC PC did not reveal promising progress until the development of a comprehensive treatment strategy including cytoreductive surgery (CRS) plus hyperthermic intraperitoneal chemotherapy (HIPEC) and perioperative chemotherapy.[8-15] This new comprehensive treatment improves the median OS of selected patients with CRC PC up to 21-63 months, and 5-year survival rate up to approximately 40% [16-28], or even 58% according to the American Society of Peritoneal Surface Malignancies (ASPSM) multi-institution study [29]. It has been widely recognized in North America, Europe, Australia, and Japan [14, 24, 26, 30-32]. In the 9th International Congress on Peritoneal Surface Malignancies in Amsterdam in 2014, peritoneal surface oncology group international (PSOGI) reached a consensus that CRS+HIPEC should be considered as the standard therapy for the selected patients with mild-to-moderate CRC PC [33].

Nevertheless, therapeutic efficacy of this comprehensive treatment strategy for CRC PC patient remains controversial due to insufficient convincing evidence. Therefore, we conducted this meta-analysis of published clinical studies to verify the efficacy of this strategy against CRC PC.

## RESULTS

### Basic characteristics of all data

#### Results of literature search

**Figure 1:**
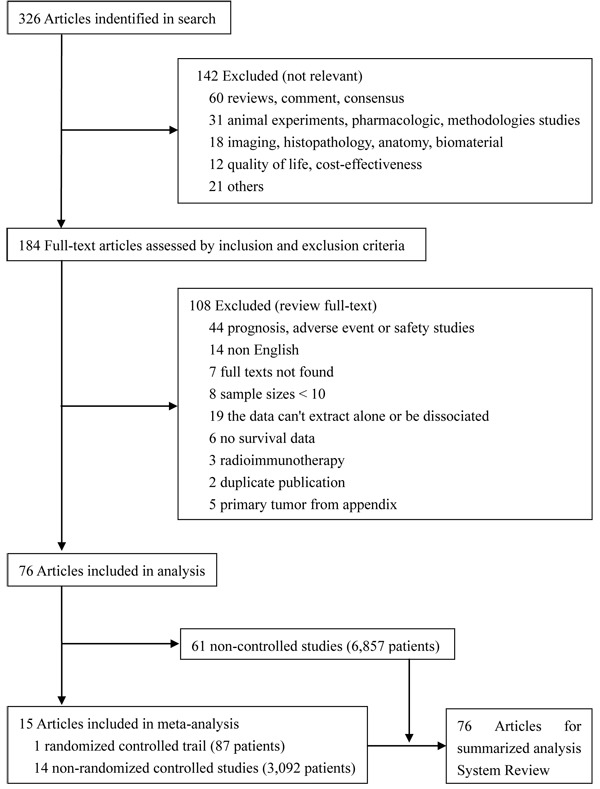
Study flowchart of systematic reviews and meta-analyses

Literature search identified 326 researches, 76 of which met the inclusion criteria, including 1 randomized controlled trail (RCT) (87 patients) [12], 14 non-randomized controlled studies (3,092 patients) [13-15, 26, 28, 29, 34-40, 99], and 61 non-controlled studies (6,857 patients) [16, 19-21, 41-92, 100-104]. The other 250 studies were excluded for miscellaneous reasons, and the flowchart of search strategy is showed in Figure 1. We conducted a meta-analysis on the 15 controlled studies (3,179 patients) and a summary of 76 HIPEC-related studies (10,036 patients).

#### Study characteristics

The characteristics of 15 controlled studies [8-15, 26, 28, 29, 34-40, 99] were shown in Table 1-5, and all 76 selected studies [12-16, 19-21, 26, 28, 29, 34-92, 99-104] were summarized in Table 6-10. All these studies were published between 1993 and 2016 as full texts, performed in 19 countries and regions (Table 11-19). Fifty-eight studies were single-center studies [12, 16, 19, 21, 35, 36, 38-43, 45, 43-53, 55-57, 60-63, 66-71, 74-83, 86-92, 99, 100, 102-104], and the other 18 were multicenter studies (participating institutions from 2 to 28) [13-15, 28, 29, 34, 37, 44, 46, 54, 58, 59, 64, 65, 72, 73, 84, 85, 101]. In these multicenter studies, 6 studies were performed by over 10 participating institutions included studies conducted by Glehen et al (*n* = 28, a central database) [13], Glehen et al (*n* = 25, a central database) [54], Elias et al (*n* = 25, a central database) [14], Esquivel et al (*n* = 21, The American Society of Peritoneal Surface Malignancies (ASPSM)) [29], and Prada-Villaverde et al (*n* = 15) [72]. A total of 63 articles were retrospective studies, in which 11 articles were included in this meta-analysis [13-16, 19-21, 28, 29, 34, 37-40, 43-48, 50-52, 54-57, 59-66, 68-72, 74-88, 91, 99-104]. Thirteen articles were prospective studies, in which 4 were included in this meta-analysis [12, 26, 35, 36, 42, 49, 53, 58, 67, 73, 89, 90, 92]. According to the North-England evidence-based guidelines [34, 35], there was one evidence level Ib in this meta-analysis [12], the rest cohort studies or “outcome” researches were evidence level II.[13-15, 26, 28, 29, 34-40, 99]

**Table 1:** Major Characteristics of Fifteen Controlled Researches on Peritoneal Carcinomatosis (PC) from Colorectal Cancer (CRC) Treated with Cytoreductive Surgery (CRS) plus Hyperthermic Intraperitoneal Chemotherapy (HIPEC) versus Surgery alone with Systemic Chemotherapy (SC) and/or Early Postoperative Intraperitoneal Chemotherapy (EPIC)

Author/ Year/ Country	Participating Institutions	Study Period	Design	Level of Evidence	Number of CRC PC	Treatment strategy	
						HIPEC group	Control group
Chua TC/ 2009/ Australia [34]	2	1997-2008	retrospective	IIb	15 (15/33)	CRS+HIPEC 7 pts; HIPEC: MMC (10-20 mg/m^2^) for 90 min at 42°C using the closed abdomen technique. No EPIC. SC: FOLFOX and Bevacizumab	SC 8 pts SC: FOLFOX and Bevacizumab No HIPEC No EPIC
Chua TC/ 2011/ Australia [15]	3	1988-2009	retrospective	IIa	294 (294/294)	CRS+HIPEC+SC 110 pts HIPEC: MMC (10-20 mg/m^2^) for 90 min at 42°C using the Coliseum technique. No EPIC SC: 5-FU + LV; 5-FU + LV or CBP with L-OHP or CPT-11; or Regimen 2 + BEV, C225, or PAN	Surgery and/or SC 184 pts SC: 5-FU + LV; 5-FU + LV or CBP with L-OHP or CPT-11; or Regimen 2 + BEV, C225, or PAN No EPIC No HIPEC
Chua TC/ 2013/ Australia [26]	1	1996-2011	prospective	IIa	75 (75/98)	CRS+HIPEC with/without EPIC 75pts HIPEC: MMC (10–12.5 mg/m^2^) or L-OHP (460 mg/m^2^) for 90 min at 42°C using the closed abdomen technique; Before starting HIEPC, oxaliplatin, 5-FU (400 mg/m^2^) and LV (20 mg/m^2^) by intravenous perfusion. EPIC: 5-FU (650–800 mg/m^2^/d) on Day 1-5 after surgery SC (not reported)	EPIC alone 23 pts EPIC: 5-FU (650–800 mg/m^2^/d) on Day 1-5 after surgery SC (not reported) No HIPEC

Note: CRC: colorectal cancer; PC: peritoneal carcinomatosis; Pts: patients; MMC: mitomycin C; DDP: cisplatin; FU: fluorouracil; LV: leucovorin; L-OHP: oxaliplatin; CPT-11: irinotecan; Cap: capecitabine; C225: cetuximab; CPT: camptothecin; BEV: bevacizumab; DXL: docetaxel; CBP: carboplatin; PAN: panitumumab;

**Table 2:** Major Characteristics of Fifteen Controlled Researches on Peritoneal Carcinomatosis (PC) from Colorectal Cancer (CRC) Treated with Cytoreductive Surgery (CRS) plus Hyperthermic Intraperitoneal Chemotherapy (HIPEC) versus Surgery alone with Systemic Chemotherapy (SC) and/or Early Postoperative Intraperitoneal Chemotherapy (EPIC)

Author/ Year/ Country	Participating Institutions	Study Period	Design	Level of Evidence	Number of CRC PC	Treatment strategy	
						HIPEC group	Control group
Elias D/ 2001/ France [35]	1	1993-1999	prospective	IIa	55 (55/64)	HIPEC 27 pts HIPEC: 1. MMC (5, 8, or 10 mg/L) for 1 h between 41 °C and 44 °C using the Coliseum technique. 2. MMC (20 mg/m^2^) + DDP (200 mg/m^2^) for 1 h between 41 °C and 44 °C using the Coliseum technique. EPIC: MMC (10 g/m^2^) on Day 1 + 5-FU (500 mg/m^2^) form Day 2 to Day 6 lasted 23 h No SC	EPIC37 pts EPIC: MMC (10 g/m^2^) on Day 1 + 5-FU (500 mg/m^2^) form Day 2 to Day 6 lasted 23 h No HIPEC No SC
Elias D/ 2007/ France [36]	1	1999-2002 1994-2000	prospective	IIa	46 (46/46)	CRS+HIPEC 23 pts HIPEC: L-OHP (460 mg/m^2^) for 35 min between 42-44°C using the Coliseum technique; Before starting HIEPC, 5-FU (400 mg/m^2^) and LV (20 mg/m^2^) by intravenous perfusion. EPIC: MMC (10 mg/m^2^) at day 0, then 5-FU (650 mg/m^2^) for the next 4days SC (not reported)	EPIC 23 pts EPIC: MMC (10 mg/m^2^) at day 0, then 5-FU (650 mg/m^2^) for the next 4days SC (not reported) No HIPEC
Elias D/ 2009/ France [28]	6 (Only one centre conducted HIPEC, the rest of 5 as a control)	1998-2003	retrospective	IIa	96 (96/96)	Neoadjuvant IPC+CRS+HIPEC+SC 48 pts Neoadjuvant IPC: L-OHP or CPT-11 (not reported the detailed regimen) HIPEC: L-OHP (460 mg/m^2^) over 30 min at 43°C using the Coliseum technique. Before starting HIEPC, 5-FU 400 mg/m^2^ and LV 20 mg/m^2^ by intravenous perfusion. SC: 1. FU Plus CPT-11 or L-OHP, LV; 2. Cap Plus L-OHP; 3. CPT-11 plus C225 and CPT	Surgery and/or SC 48 pts SC: 1. FU Plus CPT-11 or L-OHP, LV; 2. Cap Plus L-OHP; 3. CPT-11 plus C225 and CPT No HIPEC No EPIC

Note: CRC: colorectal cancer; PC: peritoneal carcinomatosis; Pts: patients; MMC: mitomycin C; DDP: cisplatin; FU: fluorouracil; LV: leucovorin; L-OHP: oxaliplatin; CPT-11: irinotecan; Cap: capecitabine; C225: cetuximab; CPT: camptothecin; BEV: bevacizumab; DXL: docetaxel; CBP: carboplatin; PAN: panitumumab;

**Table 3:** Major Characteristics of Fifteen Controlled Researches on Peritoneal Carcinomatosis (PC) from Colorectal Cancer (CRC) Treated with Cytoreductive Surgery (CRS) plus Hyperthermic Intraperitoneal Chemotherapy (HIPEC) versus Surgery alone with Systemic Chemotherapy (SC) and/or Early Postoperative Intraperitoneal Chemotherapy (EPIC)

Author/ Year/ Country	Participating Institutions	Study Period	Design	Level of Evidence	Number of CRC PC	Treatment strategy	
						HIPEC group	Control group
Elias D/ 2010/ France [14]	25 (a central database)	1990-2007	retrospective	IIa	523 (523/523)	CRS+HIPEC with/without SC 443 pts CRS+HIPEC+EPIC with/without SC 9 pts HIPEC: 1. MMC (30-50 mg/m^2^) ± DDP (50-100 mg/m^2^) during 60 to 120min at 41°C using Coliseum or closed abdomen technique; 2. L-OHP (360-460 mg/m^2^)±CPT-11 (200 mg/m^2^) +intravenous 5-FU and LV during 30 minutes at 43°C using Coliseum or closed abdomen technique. EPIC: MMC (10 g/m^2^) on Day 1+5-FU (600 mg/m^2^) form Day 2 to Day 6 lasted 23 h SC: not reported the detailed regimen	CRS+EPIC with/without SC 84 pts EPIC: MMC (10 g/m^2^) on Day 1+5-FU (600 mg/m^2^) form Day 2 to Day 6 lasted 23 h SC: not reported the detailed regimen No HIPEC
Esquivel J/ 2014 / America [29]	21 (The American Society of Peritoneal Surface Malignancies (ASPSM))	1985-2012	retrospective	IIa	1,013 (1,013/1,013)	CRS+HIPEC 705 pts HIPEC: The chemotherapy drugs L-OHP or MMC or others but not reported the remaining details. SC (not detailed reported) No EPIC	SC alone 308 pts SC (not detailed reported) No EPIC No HIPEC
Franko J/ 2010/ America [37]	3 (one centre conducted HIPEC, two centre as a control)	2001-2007	retrospective	IIa	105 (105/105)	CRS+HIPEC+SC 67 pts HIPEC: MMC 30mg for the first hour, followed by an additional 10 mg for 40 more minutes using the closed abdomen technique. (Perfusion fluid temperature not reported) No EPIC SC: 1. 5-FU and CPT-11; 2. L-OHP or biological agents (BEV and/or C225)	Surgery + SC 38 pts SC: 1. 5-FU and CPT-11; 2. L-OHP or biological agents (BEV and/or C225) No EPIC No HIPEC

Note: CRC: colorectal cancer; PC: peritoneal carcinomatosis; Pts: patients; MMC: mitomycin C; DDP: cisplatin; FU: fluorouracil; LV: leucovorin; L-OHP: oxaliplatin; CPT-11: irinotecan; Cap: capecitabine; C225: cetuximab; CPT: camptothecin; BEV: bevacizumab; DXL: docetaxel; CBP: carboplatin; PAN: panitumumab;

**Table 4:** Major Characteristics of Fifteen Controlled Researches on Peritoneal Carcinomatosis (PC) from Colorectal Cancer (CRC) Treated with Cytoreductive Surgery (CRS) plus Hyperthermic Intraperitoneal Chemotherapy (HIPEC) versus Surgery alone with Systemic Chemotherapy (SC) and/or Early Postoperative Intraperitoneal Chemotherapy (EPIC)

Author/ Year/ Country	Participating Institutions	Study Period	Design	Level of Evidence	Number of CRC PC	Treatment strategy	
						HIPEC group	Control group
Gervais MK/ 2013/ Canada [38]	1	2004-2011	retrospective	IIa	40 (40/40)	Neoadjuvant SC with/without neoadjuvant radiotherapy+ CRS+HIPEC+SC 25 pts Neoadjuvant SC: BEV HIPEC: L-OHP (460 mg/m^2^) for 30 min between 42-44°C using the Coliseum technique; Before starting HIEPC, 5-FU (400 mg/m^2^) and LV (20 mg/m^2^) by intravenous perfusion. No EPIC SC: 5-FU, LV, L-OHP, and/or CPT-11, with or without BEV	Neoadjuvant SC with/without neoadjuvant radiotherapy + surgery + SC 15 pts Neoadjuvant SC: BEV SC: 5-FU, LV, L-OHP, and/or CPT-11, with or without BEV No EPIC No HIPEC
Glehen O/ 2004/ France [13]	28 (a central database)	1987-2002	retrospective	IIa	506 (506/506)	CRS+HIPEC with/without SC 383 pts CRS+HIPEC with/without EPIC/SC 112 pts HIPEC: MMC/MMC+DDP, L-OHP, MMC+CPT-11, 5-FU, others during 30 to 90 min at 40-43°C using Coliseum or closed abdomen technique. EPIC: 5-FU (15 mg/kg/d) on Day 1-5 after surgery SC: 1. 5-FU + LV with/without DDP/L-OHP; 2. 5-FU alone; 3. 5-FU + LV+ L-OHP+ CPT-11; 4. Others and unknown	CRS+EPIC with/without SC 235 pts EPIC: 5-FU (15 mg/kg/d) on Day 1-5 after surgery SC: 1. 5-FU + LV with/without DDP/L-OHP; 2. 5-FU alone; 3. 5-FU + LV+ L-OHP+ CPT-11; 4. Others and unknown No HIPEC
Goéré D/ 2015/ France [99]	1	2000-2010	retrospective	IIa	139 (139/180)	Neoadjuvant SC +CRS+HIPEC+SC with/without EPIC 139 pts HIPEC: L-OHP+CPT-11 (72%), CPT-11 alone (15%), other items not reported. SC: 1. 5-FU + L-OHP; 2. 5-FU + CPT-11; 3. 5-FU alone EPIC: MMC/5-FU	Neoadjuvant SC +Surgery+SC 41 pts SC: 1. 5-FU + L-OHP; 2. 5-FU + CPT-11; 3. 5-FU alone

Note: CRC: colorectal cancer; PC: peritoneal carcinomatosis; Pts: patients; MMC: mitomycin C; DDP: cisplatin; FU: fluorouracil; LV: leucovorin; L-OHP: oxaliplatin; CPT-11: irinotecan; Cap: capecitabine; C225: cetuximab; CPT: camptothecin; BEV: bevacizumab; DXL: docetaxel; CBP: carboplatin; PAN: panitumumab;

**Table 5:** Major Characteristics of Fifteen Controlled Researches on Peritoneal Carcinomatosis (PC) from Colorectal Cancer (CRC) Treated with Cytoreductive Surgery (CRS) plus Hyperthermic Intraperitoneal Chemotherapy (HIPEC) versus Surgery alone with Systemic Chemotherapy (SC) and/or Early Postoperative Intraperitoneal Chemotherapy (EPIC)

Author/ Year/ Country	Participating Institutions	Study Period	Design	Level of Evidence	Number of CRC PC	Treatment strategy	
						HIPEC group	Control group
Huang CQ/ 2014/ China [39]	1	2004-2013	retrospective	IIa	62 (62/62)	CRS+HIPEC+SC with/without PIC 33 pts HIPEC: MMC (30 mg) + DDP (120 mg) for 90 min at 43.0±0.5°C using the Coliseum technique EPIC: DXL (75 mg/m^2^, on day 1, every 3 weeks) and CBP (at Calvert formula: area under the curve, AUC 5; on day 1, every 3 weeks) SC: FOLFOX or FOLFIRI	CRS+ SC with/without PIC 29 pts SC: FOLFOX or FOLFIRI EPIC: DXL (75 mg/m^2^, on day 1, every 3 weeks) and CBP (at Calvert formula: AUC 5; on day 1, every 3 weeks) No HIPEC
Passot G/ 2014/ France [40]	1	2005-2012	retrospective	IIa	82 (82/115)	Neoadjuvant SC+CRS+HIPEC 82 pts Neoadjuvant SC: 1. FOLFIRI with/without BEV or C225; 2. FOLFOX with/without BEV or C225; 3. Others regimens. HIPEC: L-OHP (360 mg/m^2^) for 30 min using the closed abdomen technique, not reported the perfusion temperature. No EPIC No SC	Neoadjuvant SC + Surgery + SC 33 pts Neoadjuvant SC: 1. FOLFIRI with/without BEV or C225; 2. FOLFOX with/without BEV or C225; 3. Others regimens. No EPIC SC (uncertainty)
Verwaal VJ/ 2003 /Netherlands [12]	1	1998-2001	prospective	Ib	87 (87/105)	CRS+HIPEC with/without SC 54 pts HIPEC: MMC (17.5 mg/m^2^) for 90 min between 42-44°C using the Coliseum technique No EPIC SC: 1. 5-FU (400 mg/m^2^) + LV (80 mg/m^2^); 2. FU + CPT-11 (350 mg/m^2^)	Surgery and/or SC 51 pts SC: 1. 5-FU (400 mg/m^2^) + LV (80 mg/m^2^); 2. FU + CPT-11 (350 mg/m^2^) No EPIC No HIPEC

Note: CRC: colorectal cancer; PC: peritoneal carcinomatosis; Pts: patients; MMC: mitomycin C; DDP: cisplatin; FU: fluorouracil; LV: leucovorin; L-OHP: oxaliplatin; CPT-11: irinotecan; Cap: capecitabine; C225: cetuximab; CPT: camptothecin; BEV: bevacizumab; DXL: docetaxel; CBP: carboplatin; PAN: panitumumab;

**Table 6:** Major Characteristics of Sixty-one Single Arm Researches on Peritoneal Carcinomatosis (PC) from Colorectal Cancer (CRC) Treated with Cytoreductive Surgery (CRS) plus Hyperthermic Intraperitoneal Chemotherapy (HIPEC)

Author/ Years/ Country	Participating Institutions	Study Period	Design	Level of evidence	Number of CRC PC	HIPEC
Alzahrani/ 2015/ Australia [41]	1	1996-2014	retrospective	III	205 (205/675)	Before HIPEC, 5-FU (400 mg/m^2^) were delivered by systemic i.v., L-OHP (350 mg/m^2^) for 30 min at 43°C using coliseum technique.
Beaujard/ 2000/ France [42]	1	1991-1997	prospective	IIb	27 (27/86)	MMC (10 mg/L) for 90 min at inflow temperature 46-49 °C using the closed abdomen technique.
Bijelic/ 2008/ Australia [43]	1	1981-2004	retrospective	III	70 (70/472)	MMC (10 mg/m^2^ for females and 12.5 mg/m^2^ for males) for 90 min at about 42 °C using the coliseum technique.
Braam/ 2014/ Australia [44]	2	2005-2013	retrospective	III	132 (132/132)	MMC (17.5 mg/m^2^ an additional 8.8 mg/m^2^ at an interval of 30 and 60 min) for 90 min at 42 °C using the coliseum technique.
Cao/ 2009/ Australia [45]	1	1995-2008`	retrospective	III	52 (52/467)	MMC (10-12.5 mg/m^2^) for 90 min at 42 °C using coliseum technique.
Cavaliere/ 2006/ Italy [46]	6	1996-2005	retrospective	III	120 (120/120)	MMC (3.3 mg/m^2^/L) + DDP (25 mg/m^2^/L) for 60-90 min at 41.5-43 °C using the coliseum or closed abdomen technique. After intravenous administration of 5-FU (400 mg/m^2^) and LV (20 mg/m^2^), L-OHP (460 mg/m^2^) for 30 min at 43 °C using the coliseum or closed abdomen technique.
Ceelen/ 2014/ Belgium [47]	1	2002-2012	retrospective	III	152 (152/166)	Before HIPEC, LV (20 mg/m^2^) and 5-FU (400 mg/m^2^) were delivered by systemic i.v. L-OHP (460 mg/m^2^) or MMC (35 mg/m^2^) for 30-90 min at 41 °C using coliseum technique.
Desantis/ 2014/ Franc^e^ [48]	1	1999-2011	retrospective	III	74 (74/356)	MMC (10 mg/m^2^ for females and 12.5 mg/m^2^ for males) for 90 min at 43°C using coliseum or closed abdomen technique.
Elias/ 2004/ France [49]	1	1998-2001	prospective	IIb	24 (24/24)	One hour before HIPEC, LV (20 mg/m^2^) and 5-FU (400 mg/m2) were delivered by systemic i.v. HIPEC: L-OHP (460 mg/m^2^) for 30 min at 43 °C using the coliseum technique.
Elias/ 2014/ France [50]	1	1995-2010	retrospective	III	119 (119/443)	MMC (5, 8, or 10 mg/L) for 1 h between 41 °C and 44 °C using the coliseum technique. MMC (20 mg/m^2^) + DDP (200 mg/m^2^) for 1 h between 41 °C and 44 °C using the coliseum technique. L-OHP 460 mg/m^2^ over 30 min at 43°C using the coliseum technique. MMC (12.9+/-3.8 mg/m^2^) for 90 min between 41 °C and 42 °C using closed abdomen technique.

Note: MMC: mitomycin C; DDP: cisplatin; FU: fluorouracil; LV: leucovorin; L-OHP: oxaliplatin; CPT-11: irinotecan; NR: not reported

**Table 7:** Major Characteristics of Sixty-one Single Arm Researches on Peritoneal Carcinomatosis (PC) from Colorectal Cancer (CRC) Treated with Cytoreductive Surgery (CRS) plus Hyperthermic Intraperitoneal Chemotherapy (HIPEC)

Author/ Years/ Country	Participating Institutions	Study Period	Design	Level of evidence	Number of CRC PC	HIPEC
Evers/ 2011/ Netherlands [51]	1	2001-2009	retrospective	III	108 (108/194)	MMC (35 mg/m^2^) for 90 min at 40-41 °C, perfusion mode not reported.
Faron M/ 2016/ France [100]	1	2003-2012	retrospective	III	173 (173/173)	Before HIPEC, LV (20 mg/m^2^) and 5-FU (400 mg/m2) were delivered by systemic i.v. HIPEC: L-OHP (300 mg/m^2^) and CPT-11 (200 mg/m^2^) for 30 min between 43 °C using closed abdomen technique.
Franko/ 2008/ America [52]	1	2001-2007	retrospective	III	65 (65/65)	MMC (40 mg/m^2^) for 90 min using closed abdomen technique. (have not reported the liquid perfusion temperature)
Frøysnes/ 2016/ Norway[103]	1	2004-2013	retrospective	III	119 (119/144)	MMC (35 mg/m^2^) for 90 min between 39.5 °C and 41.2 °C using closed abdomen technique until 2008, and thereafter a closed technique with open abdomen
Glehen/ 2003/ France [53]	1	1998-2001	prospective	IIb	26 (26/56)	MMC (0.7 mg/kg) for 90 min at 46-48 °C using closed abdomen technique.
Glehen/ 2004/ France [16]	1	1989-2002	retrospective	III	53 (53/53)	MMC (total dose 40-60 mg) for 90 min at 46-48 °C using closed abdomen technique.
Glehen/ 2010/ France [54]	25	1989-2007	retrospective	III	523 (523/1290)	MMC (30-50 mg/m^2^) with or without DDP (50-100 mg/m^2^) for 60-120 min at 41-42.5 °C using the coliseum or closed abdomen technique. L-OHP (360-460 mg/m^2^) with or without CPT-11 (100-200 mg/m^2^) with or without intravenous 5-FU and LV delivered over 30 min at 43°C using the coliseum or closed abdomen technique.
Gomes da Silva/ 2005/ America [55]	1	1981-2004	retrospective	III	11 (11/11)	MMC (10 mg/m^2^ in females and 12.5 mg/m^2^ in males) for 90 min at 41-42 °C using closed abdomen technique.
Gusani/ 2008/ America [56]	1	2002-2005	retrospective	III	28 (25/122)	MMC (30 mg) for 60 min at 40-42 °C using closed abdomen technique, after 60 min, additional MMC (10 mg) was added for 40 more min.
Hamilton/ 2011/ Canada [57]	1	2000-2008	retrospective	III	31 (31/101)	MMC (12-15 mg) for 90 min at 40-42 °C using coliseum technique.
Hompes/ 2012/ Belgium [58]	6	2004-2008	prospective	IIb	39 (39/48)	L-OHP (460 mg/m^2^) for 30 min at 41-42 °C using coliseum technique. Before HIPEC, systemic LV (20 mg/m^2^) and 5-FU (400 mg/m^2^) were administered.

Note: MMC: mitomycin C; DDP: cisplatin; FU: fluorouracil; LV: leucovorin; L-OHP: oxaliplatin; CPT-11: irinotecan; NR: not reported

**Table 8:** Major Characteristics of Sixty-one Single Arm Researches on Peritoneal Carcinomatosis (PC) from Colorectal Cancer (CRC) Treated with Cytoreductive Surgery (CRS) plus Hyperthermic Intraperitoneal Chemotherapy (HIPEC)

Author/ Years/ Country	Participating Institutions	Study Period	Design	Level of evidence	Number of CRC PC	HIPEC
Hompes/ 2014/ Belgium [59]	2	2004-2006 2006-2010	retrospective	IIb	95 (95/95)	MMC (35 mg/m^2^) for 90 min at 41-42 °C using coliseum or closed abdomen technique. Before HIPEC, LV (20 mg/m^2^) and 5-FU (400 mg/m^2^) were delivered by systemic i.v. L-OHP (460 mg/m^2^) for 30 min at 41-42 °C using coliseum or closed abdomen technique.
Iversen/ 2013/ Denmark [60]	1	2006-2012	retrospective	III	34 (34/80)	MMC (35 mg/m^2^) for 90 min at 41.0-42.5 °C using coliseum technique.
Kecmanovic/ 2005/ Serbia and Montenegro [61]	1	1996-2003	retrospective	III	18 (18/18)	MMC (12.5 mg/m^2^, max. 25 mg for males; 10.0 mg/m^2^, max. 20 mg for females) for 120 min at 42 °C using closed abdomen technique
Kianmanesh/ 2007/ France [62]	1	1992-2005	retrospective	III	43 (43/43)	MMC (120 mg) + DDP (200 mg/m^2^) for 90-120 min at 47-50 °C using coliseum or closed abdomen technique.
Klaver/ 2011/ Netherlands [63]	1	1997-2008	retrospective	III	21 (21/21)	MMC (35 mg/m^2^) for 90 min at 41°C using coliseum technique.
Klaver/ 2012/ Netherlands [64]	2	1996-2010	retrospective	III	17 (17/24)	MMC or L-OHP for 90 min at 42°C using coliseum technique.
Kuijpers/ 2013/ Netherlands [65]	6	1995-2012	retrospective	III	660 (660/960)	MMC (35 mg/m^2^) (in three fractions (one half, one fourth, and one fourth of the total dose)) for 90 min at 41-42 °C using coliseum technique.
Kuijpers/ 2014/ Netherlands [66]	1	2004-2012	retrospective	III	73 (73/73)	MMC (35 mg/m^2^) for 90 min at 41-42 °C using coliseum technique.
Lanuke/ 2009/ Canada [67]	1	2000-2008	prospective	IIb	31 (31/101)	MMC (12-15 mg) for 60 min at 40-42 °C using coliseum technique.
Levine/ 2014/ America [68]	1	1991-2013	retrospective	III	232 (232/1000)	MMC (30 mg) for 60-90 min at 38.5-43 °C using coliseum technique; L-OHP (200 mg/m^2^) for selected patients.
Maillet M/ 2016/ France [101]	4	2004-2012	retrospective	III	231 (231/231)	NR
McConnell/ 2013/ Canada [69]	1	2000-2011	retrospective	III	245 (245/245)	MMC (12-15 mg) for 60 min at 40-42 °C using coliseum or closed abdomen technique. L-OHP (400 mg/m^2^) for 60 min at 40-42 °C using coliseum or closed abdomen technique with a simultaneous dose of intravenous 5-FU (800 mg)..
Nikolic/ 2014/ Serbia [70]	1	2005-2012	retrospective	III	61 (61/61)	L-OHP (410 mg/m^2^) for 30-60 min at 41 °C using closed abdomen technique.

Note: MMC: mitomycin C; DDP: cisplatin; FU: fluorouracil; LV: leucovorin; L-OHP: oxaliplatin; CPT-11: irinotecan; NR: not reported

**Table 9:** Major Characteristics of Sixty-one Single Arm Researches on Peritoneal Carcinomatosis (PC) from Colorectal Cancer (CRC) Treated with Cytoreductive Surgery (CRS) plus Hyperthermic Intraperitoneal Chemotherapy (HIPEC)

Author/ Years/ Country	Participating Institutions	Study Period	Design	Level of evidence	Number of CRC PC	HIPEC
Passot/ 2012/ France [21]	1	1991-2010	retrospective	III	120 (120/120)	MMC (10 mg/ml, total dose 40-60mg) for 90 min at 46-48 °C using closed abdomen technique. MMC (0.7 mg/kg) + CPT-11 (100 mg/m^2^) for 90 min at 44-46 °C using closed abdomen technique. MMC (30-50 mg/m^2^) with or without DDP (50-100 mg/m^2^) for 60-120 min at 41-42.5 °C using coliseum technique or closed abdomen technique. L-OHP (360-460 mg/m^2^) with or without CPT-11 (100-200 mg/m^2^) with or without intravenous 5-FU and LV for 30 min at 43°C using coliseum technique or closed abdomen technique.
Passot/ 2016/ France [104]	1	1989-2015	retrospective	III	342 (342/1,125)	Idem (Passot/ 2012/ France [21])
Pilati/ 2003/ Italy [71]	1	1995-2001	retrospective	III	46 (46/46)	MMC (3.3 mg/m^2^/L) with or without DDP (25 mg/m^2^/L) for 90 min at 41.2-42.1 °C using coliseum technique or closed abdomen technique.
Prada-Villaverde/ 2014/ Spai^n^ [72]	15	2000-2011	retrospective	III	539 (539/539)	MMC or L-OHP for 30-120 min at 40-43°C using coliseum or closed abdomen technique.
Quenet/ 2011/ France [73]	2	1998-2007 2002-2007	prospective	IIb	146 (146/146)	L-OHP (460 mg/m^2^) with intravenous 5-FU (400 mg/m^2^) and LV (20 mg/m^2^) for 30 min at 42-45 °C using coliseum technique. L-OHP (300 mg/m^2^) with CPT-11 (200 mg/m^2^) with intravenous 5-FU (400 mg/m^2^) and LV (20 mg/m^2^) for 30 min at 42-45 °C using coliseum technique.
Rivard/ 2014/ Canada [74]	1	2003-2011	retrospective	III	68 (68/68)	NR
Rodt/ 2013/ Denmark [75]	1	2006-2011	retrospective	III	19 (19/35)	NR
Shen/ 2004/ America [20]	1	1991-2002	retrospective	III	77 (77/77)	MMC (total dose 30 mg) for 60-120 min at 38.5-43 °C using closed abdomen technique.
Shen/ 2008/ America [76]	1	1992-2005	retrospective	III	55 (55/150)	MMC (total dose 30 mg) for 60-120 min at 38.5-43 °C using closed abdomen technique.
Simkens GA/ 2015/ Netherlands [102]	1	2007-2013	retrospective	III	133 (133/133)	MMC (35 mg/m^2^) for 90 min at 41.1 °C using open-coliseum technique.
Swellengrebel/ 2009/ Netherlands [77]	1	1999-2005	retrospective	III	92 (92/92)	MMC (35 mg/m^2^) for 90 min at 41-42 °C using coliseum technique.

Note: MMC: mitomycin C; DDP: cisplatin; FU: fluorouracil; LV: leucovorin; L-OHP: oxaliplatin; CPT-11: irinotecan; NR: not reported

**Table 10:** Major Characteristics of Sixty-one Single Arm Researches on Peritoneal Carcinomatosis (PC) from Colorectal Cancer (CRC) Treated with Cytoreductive Surgery (CRS) plus Hyperthermic Intraperitoneal Chemotherapy (HIPEC)

Author/ Years/ Country	Participating Institutions	Study Period	Design	Level of evidence	Number of CRC PC	HIPEC
Tabrizian/ 2014/ America [78]	1	2007-2012	retrospective	III	51 (51/170)	MMC (total dose 40 mg) for 90 min at 41-43 °C using closed abdomen technique.
Teo/ 2013/ Singapore [79]	1	2001-2012	retrospective	III	28 (28/100)	MMC for 60 min at 42 °C using closed abdomen technique.
Teo/ 2014/ Singapore [80]	1	2001-2012	retrospective	III	35 (35/35)	MMC for 60 min at 42 °C using closed abdomen technique.
Ung/ 2013/ Australia [81]	1	2000-2012	retrospective	III	125 (125/211)	MMC (12.5 mg/m^2^) for 90 min at 42 °C using coliseum technique.
Vaira/ 2010/ Italy [82]	1	2002-2008	retrospective	III	40 (40/72)	MMC (16 mg/m^2^) + DDP (100 mg/m^2^) for 60 min at 41.5 °C using semi-closed abdomen technique. Before HIPEC, LV (20 mg/m^2^) and 5-FU (400 mg/m^2^) were delivered by systemic i.v. L-OHP (460 mg/m^2^) for 30 min at 42 °C using semi-closed abdomen technique.
van Leeuwen / 2008/ Sweden [83]	1	2003-2006	retrospective	III	38 (38/103)	Before HIPEC, LV (30 mg/m^2^) and 5-FU (500 mg/m^2^) were delivered by systemic i.v. HIPEC: L-OHP (460 mg/m^2^) for 30 min at 42-44 °C using the coliseum technique.
van Oudheusden/ 2014/ Netherlands [84]	2	2005-2013	retrospective	III	113 (113/149)	MMC (35 mg/m^2^) for 90 min at 41-42 °C using coliseum technique.
van Oudheusden / 2015/ Netherlands [85]	2	2005-2013	retrospective	III	252 (252/351)	MMC (35 mg/m^2^) for 90 min at 41.1 °C using open-coliseum technique.
Varban/ 2009/ America [86]	1	1991-2007	retrospective	III	128 (128/142)	MMC (total dose 30 mg) for 60 or 90 min at 42.5 °C using closed abdomen technique. MMC (total dose 40 mg) for 120 min at 42.5 °C using closed abdomen technique.
Verwaal/ 2005/ Netherlands [19]	1	1995-2003	retrospective	III	117 (117/117)	MMC (35 mg/m^2^) for 90 min at 40-41 °C using coliseum technique.
Votanopoulos/ 2013/ America [87]	1	1993-2011	retrospective	III	217 (217/217)	MMC for 90-120 min at 40.5-43 °C using closed abdomen technique.
Winer/ 2014/ America [88]	1	2001-2011	retrospective	III	30 (30/67)	MMC (total dose 40 mg) for 100 min at 42 °C using closed abdomen technique.
Witkamp/ 2001/ Netherlands [89]	1	1995-1997	prospective	IIb	29 (29/29)	MMC (15-40 mg/m^2^ initially; 35 mg/m^2^ majority) for 90 min at 40-41 °C using closed abdomen technique.
Yan/ 2006/ Australia [90]	1	1997-2006	prospective	IIb	30 (30/30)	MMC (10-12.5 mg/m^2^) for 90 min at 42 °C using coliseum technique.
Yan/ 2008/ Australia [91]	1	1997-2007	retrospective	III	50 (50/50)	MMC (10-12.5 mg/m^2^) for 90 min at 42 °C using coliseum technique.
Zanon/ 2006/ Italy [92]	1	1998-2004	prospective	III	25 (25/25)	MMC (15 mg/m^2^) for 60 min at 42 °C using closed abdomen technique.

Note: MMC: mitomycin C; DDP: cisplatin; FU: fluorouracil; LV: leucovorin; L-OHP: oxaliplatin; CPT-11: irinotecan; NR: not reported

**Table 11:** Summary of HIEPC-related procedures in different PC institutions or countries (published researches)

Country /No. Institutions	Major Institutions	No. patients	Mode	HIPEC-MMC alone	HIPEC-MMC+DDP	HIPEC-L-OHP alone	HIPEC-other	Temperature (°C)	Duration (min)
USA, 17	Wake Forest University of Baptist Medical Center [13, 20, 68, 76, 86, 87]								
	Subtotal/Median/Range	>709	C	30 mg				40.75 (38.5-43)	90 (60-90)
	University of Pittsburgh Medical Center (University of Pittsburgh) [37, 52, 56, 88]								
	Subtotal/Median/Range	190	C	40 mg				42 (40-42)	100 (90-100)
	Washington Hospital Center [13, 43, 55]								
	Subtotal/Median/Range	>81	C	10 or 12.5 mg/m^2^				42 (40-43)	90 (30-90)
	Cancer Treatment Centers of America [29, 72]								
	Subtotal/Median/Range	?	O/C	Y		Y		40-43	30-120
	Loma Linda University Medical Center [29, 72]								
	Subtotal/Median/Range	?	O/C	Y		Y		40-43	30-120
	Medical College of Wisconsin [29, 72]								
	Subtotal/Median/Range	?	O/C	Y		Y		40-43	30-120
	Mercy Medical Center [29, 72]								
	Subtotal/Median/Range	?	O/C	Y		Y		40-43	30-120
	Moores Cancer Center, University of California [29, 72]								
	Subtotal/Median/Range	?	O/C	Y		Y		40-43	30-120
	Rutgers University [29, 72]								
	Subtotal/Median/Range	?	O/C	Y		Y		40-43	30-120
	St Agnes Hospital [15, 34]								
	Subtotal/Median/Range	>30	O/C	10-20 mg/m^2^				42	90

Note: C: closed abdomen technique for HIPEC; O: open abdomen technique for HIPEC; Y: yes; MMC: mitomycin C; DDP: cisplatin; 5-FU: fluorouracil; L-OHP: oxaliplatin; CPT-11: irinotecan

**Table 12:** Summary of HIEPC-related procedures in different PC institutions or countries (published researches)

Country /No. Institutions	Major Institutions	No. patients	Mode	HIPEC-MMC alone	HIPEC-MMC+DDP	HIPEC-L-OHP alone	HIPEC-other	Temperature (°C)	Duration (min)
USA, 17	St. John Hospital [29, 72]								
	Subtotal/Median/Range	?	O/C	Y		Y		40-43	30-120
	Tufts Medical Center [29, 72]								
	Subtotal/Median/Range	?	O/C	Y		Y		40-43	30-120
	University of Illinois [29, 72]								
	Subtotal/Median/Range	?	O/C	Y		Y		40-43	30-120
	University of Miami [29, 72]								
	Subtotal/Median/Range	?	O/C	Y		Y		40-43	30-120
	American Society of Peritoneal Surface Malignancies (ASPSM) [29]	?	NR	Y		Y	others	NR	NR
	Mount Sinai Medical Center [78]	51	C	40 mg				41-43	90
	Sharp Health Care [13]	?	O/C	Y	Y	Y	MMC+CPT-11, 5-FU	40-43	30-90
Subtotal		>1061	C	30/40 mg 10-20 mg/m2 10 or 12.5 mg/m2		Y, 200 mg/m2	MMC+CPT-11, 5-FU	42 (40-43)	90 (60-90) /30
France, 14	Centre Hospitalo-Universitaire Lyon Sud [14, 16, 21, 29, 13, 40, 42, 53, 72, 101]								
	Subtotal/Median/Range	>500	C	10 mg/L 0.7 mg/kg 40-60 mg 30-50 mg/m^2^	30-50 mg/m^2^ + 50-100 mg/m^2^	360 mg/m^2^ 360-460 mg/m^2^	MMC+CPT-11, 5-FU MMC (0.7 mg/kg) + CPT-11 (100 mg/m^2^) L-OHP (360-460 mg/m^2^) + CPT-11 (100-200 mg/m^2^)	44 (46-48) /43	90 (60-90) /30

Note: C: closed abdomen technique for HIPEC; O: open abdomen technique for HIPEC; Y: yes; MMC: mitomycin C; DDP: cisplatin; 5-FU: fluorouracil; L-OHP: oxaliplatin; CPT-11: irinotecan

**Table 13:** Summary of HIEPC-related procedures in different PC institutions or countries (published researches)

Country /No. Institutions	Major Institutions	No. patients	Mode	HIPEC-MMC alone	HIPEC-MMC+DDP	HIPEC-L-OHP alone	HIPEC-other	Temperature (°C)	Duration (min)
France, 14	Gustave Roussy Institute [13, 14, 28, 35, 36, 49, 50, 54, 73, 99-101]								
	Subtotal/Median/Range	>700	O	5, 8, or 10 mg/L 20 mg/m^2^ 12.9+/-3.8 mg/m^2^ 30-50 mg/m^2^	20 mg/m^2^ + 200 mg/m^2^ 30-50 mg/m^2^ + 50-100 mg/m^2^	460 mg/m^2^ 360-460 mg/m^2^	MMC+CPT-11, 5-FU L-OHP (360-460 mg/m^2^) + CPT-11 (100-200 mg/m^2^) L-OHP (300 mg/m^2^) + CPT-11 (200 mg/m^2^)	43 (41-44) /43	60 (60-90) /30
	Val d’Aurelle Center [13, 14, 54, 73]								
	Subtotal/Median/Range	>66	O	30-50 mg/m^2^	30-50 mg/m^2^ + 50-100 mg/m^2^	460 mg/m^2^ 360-460 mg/m^2^	L-OHP (360-460 mg/m^2^) + CPT-11 (100-200 mg/m^2^) L-OHP (300 mg/m^2^) + CPT-11 (200 mg/m^2^)	43.5 (40-43) /43	60 (60-90) /30
	Centre Hospitalo-Universitaire l’Archet [13, 14, 54]								
	Subtotal/Median/Range	>25	O/C	30-50 mg/m^2^	30-50 mg/m^2^ + 50-100 mg/m^2^	360-460 mg/m^2^	L-OHP (360-460 mg/m^2^) + CPT-11 (100-200 mg/m^2^)	41.5 (41-43) /43	60 (60-90) /30 or 60
	Paul Papin Institute [13, 14, 54]								
	Subtotal/Median/Range	>25	O/C	30-50 mg/m^2^	30-50 mg/m^2^ + 50-100 mg/m^2^	360-460 mg/m^2^	L-OHP (360-460 mg/m^2^) + CPT-11 (100-200 mg/m^2^)	41.5 (41-43) /43	60 (60-90) /30 or 60
	French Association of Surgery [14, 54]								
	Subtotal/Median/Range	?	O/C	30-50 mg/m^2^	30-50 mg/m^2^ + 50-100 mg/m^2^	360-460 mg/m^2^	L-OHP (360-460 mg/m^2^) + CPT-11 (100-200 mg/m^2^)	41 (41-43) /43	90 (60-120) /30 or 60

Note: C: closed abdomen technique for HIPEC; O: open abdomen technique for HIPEC; Y: yes; MMC: mitomycin C; DDP: cisplatin; 5-FU: fluorouracil; L-OHP: oxaliplatin; CPT-11: irinotecan

**Table 14:** Summary of HIEPC-related procedures in different PC institutions or countries (published researches)

Country /No. Institutions	Major Institutions	No. patients	Mode	HIPEC-MMC alone	HIPEC-MMC+DDP	HIPEC-L-OHP alone	HIPEC-other	Temperature (°C)	Duration (min)
France, 14	Hospital Lariboisiere [29, 72]								
	Subtotal/Median/Range	?	O/C	Y		Y		40-43	30-120
	Louis-Mourier University Hospital [62, 62, 101]								
	Subtotal/Median/Range	>250	O/C	30-50 mg/m^2^	30-50 mg/m^2^ + 50-100 mg/m^2^ 201 mg + 200 mg/m^2^	360-460 mg/m^2^	L-OHP (360-460 mg/m^2^) + CPT-11 (100-200 mg/m^2^)	42 (41-42.5) /48.5 (47-50) /30-43	90 (90-120) /60
	Centre Hospitalier de Bellevue [13]	25	O/C	Y	Y	Y	MMC+CPT-11, 5-FU	40-43	30-90
	Centre Hospitalo-Universitaire Dijon [13]	25	O/C	Y	Y	Y	MMC+CPT-11, 5-FU	40-43	30-90
	Centre Jean Perrin [13]	25	O/C	Y	Y	Y	MMC+CPT-11, 5-FU	40-43	30-90
	CHU of Nice [48]	74	O/C	10 or 12.5 mg/m^2^				43	90
	Lyon Civil Hospices, South Lyon University Hospital Center [54]	?	O/C	30-50 mg/m^2^	30-50 mg/m^2^ + 50-100 mg/m^2^	360-460 mg/m^2^	L-OHP (360-460 mg/m^2^) + CPT-11 (100-200 mg/m^2^)	41-42.5, 30-43	90/60
	Université Claude Bernard Lyon [13]	25	O/C	Y	Y	Y	MMC+CPT-11, 5-FU	40-43	30-90
Subtotal		>1038	O	30-50 mg/m2	30-50 mg/m2 + 50-100 mg/m2	360-460 mg/m2	MMC+CPT-11, 5-FU L-OHP + CPT-11 MMC + CPT-11	41.5 (40-43) /43	60 (60-90) /30 or 60
Italy, 8	National Cancer Institute of Milan [29, 46, 72]								
	Subtotal/Median/Range	?	O/C	Y	3.3 mg/m^2^/L + 25 mg/m^2^/L	460 mg/m^2^		42 (41.5-43) /43	60 (60-90) /30
	San Giuseppe Hospital [13, 46, 82]								
	Subtotal/Median/Range	>65	O/C	Y	3.3 mg/m^2^/L + 25 mg/m^2^/L 16 mg/m^2^ + 100 mg/m^2^	460 mg/m^2^	MMC+CPT-11, 5-FU	42 (41.5-43) /43	60 (60-90) /30

Note: C: closed abdomen technique for HIPEC; O: open abdomen technique for HIPEC; Y: yes; MMC: mitomycin C; DDP: cisplatin; 5-FU: fluorouracil; L-OHP: oxaliplatin; CPT-11: irinotecan

**Table 15:** Summary of HIEPC-related procedures in different PC institutions or countries (published researches)

Country /No. Institutions	Major Institutions	No. patients	Mode	HIPEC-MMC alone	HIPEC-MMC+DDP	HIPEC-L-OHP alone	HIPEC-other	Temperature (°C)	Duration (min)
Italy, 8	Regina Elena National Cancer Institute [13, 46]								
	Subtotal/Median/Range	>25	O/C	Y	3.3 mg/m^2^/L + 25 mg/m^2^/L	460 mg/m^2^	MMC+CPT-11, 5-FU	42 (41.5-43) /43	60 (60-90) /30
	University of Padua [46, 71]								
	Subtotal/Median/Range	>46	O/C	3.3 mg/m^2^/L	3.3 mg/m^2^/L + 25 mg/m^2^/L	460 mg/m^2^		42 (41.5-43) /43	90 (60-90) /33
	Istituto Nazional Tumori [13]	25	O/C	Y	Y	Y	MMC+CPT-11, 5-FU	40-43	30-90
	Ospedale di Bentivoglio [46]	?	O/C		3.3 mg/m^2^/L + 25 mg/m^2^/L	460 mg/m^2^		41.5-43/43	60-90/30
	San Camillo-Forlanini Hospital [46]	?	O/C		3.3 mg/m^2^/L + 25 mg/m^2^/L	460 mg/m^2^		41.5-43/43	60-90/30
	San Giovanni Battista Antica Sede Hospital [92]	25	C	15 mg/m^2^				42	60
Subtotal		>186	C	Y	3.3 mg/m2/L + 25 mg/m2/L	460 mg/m2	MMC+CPT-11, 5-FU	42 (41.5-43) /43	60 (60-90) /30
Belgium, 6	Jolimont Hospital [14,54, 58]								
	Subtotal/Median/Range	?	O	30-50 mg/m^2^	30-50 mg/m^2^ + 50-100 mg/m^2^	360-460 mg/m^2^ 460 mg/m^2^	L-OHP (360-460 mg/m^2^) + CPT-11 (100-200 mg/m^2^)	41.5 (41-42.5) /43	90 (60-120) /30 or 60
	Ghent University Hospital [47, 58]								
	Subtotal/Median/Range	>152	O	35 mg/m^2^		460 mg/m^2^		41 (41-42)	60 (60-90) /30
	University Hospitals Gasthuisberg [58, 59]								
	Subtotal/Median/Range	>39	O			460 mg/m^2^		41-42	30

Note: C: closed abdomen technique for HIPEC; O: open abdomen technique for HIPEC; Y: yes; MMC: mitomycin C; DDP: cisplatin; 5-FU: fluorouracil; L-OHP: oxaliplatin; CPT-11: irinotecan

**Table 16:** Summary of HIEPC-related procedures in different PC institutions or countries (published researches)

Country /No. Institutions	Major Institutions	No. patients	Mode	HIPEC-MMC alone	HIPEC-MMC+DDP	HIPEC-L-OHP alone	HIPEC-other	Temperature (°C)	Duration (min)
Belgium, 6	I-Biostat, Katholieke Universiteit Leuven and Universiteit Hasselt [58]	?	O			460 mg/m^2^		41-42	30
	UCL Mont-Godinne [58]	?	O			460 mg/m^2^		41-42	30
	Ziekenhuis Oost-Limburg [58]	?	O			460 mg/m^2^		41-42	30
Subtotal		>191	O	30-50 mg/m2	30-50 mg/m2 + 50-100 mg/m2	460 mg/m2	L-OHP + CPT-11	41 (41-42) /41-42	90 (60-90) /30 or 60
Netherlands, 6	Netherlands Cancer Institute [12, 19,51, 59, 65, 66, 77, 89]								
	Subtotal/Median/Range	863	O	35 mg/m^2^				41.5 (41-42)	90
	Catharina Hospital Eindhoven [44, 63-65, 84, 85, 102]								
	Subtotal/Median/Range	>300	O	35 mg/m^2^				41.5 (41-42)	90
	Sint Antonius Hospital Nieuwegein [44, 65, 84, 85]								
	Subtotal/Median/Range	>121	O	35 mg/m^2^				41.5 (41-42)	90
	Radboud University Nijmegen Medical Center [64]	12	O	35 mg/m^2^				41-42	90
	University Medical Center Groningen [64]	48	O	35 mg/m^2^				41-42	90
	VU Medical Centre Amsterdam [64]	17	O	35 mg/m^2^				41-42	90
Subtotal		>1432	O	35 mg/m2				41.5 (41-42)	90
Spain 6	Hospital San Jaime [29, 71]								
	Subtotal/Median/Range	?	O/C	Y		Y		40-43	30-120
	Hospital Torrecardenas [29, 71]								
	Subtotal/Median/Range	?	O/C	Y		Y		40-43	30-120

Note: C: closed abdomen technique for HIPEC; O: open abdomen technique for HIPEC; Y: yes; MMC: mitomycin C; DDP: cisplatin; 5-FU: fluorouracil; L-OHP: oxaliplatin; CPT-11: irinotecan

**Table 17:** Summary of HIEPC-related procedures in different PC institutions or countries (published researches)

Country /No. Institutions	Major Institutions	No. patients	Mode	HIPEC-MMC alone	HIPEC-MMC+DDP	HIPEC-L-OHP alone	HIPEC-other	Temperature (°C)	Duration (min)
Spain, 6	M. D. Anderson Cancer Center [29, 71]								
	Subtotal/Median/Range	?	O/C	Y		Y		40-43	30-120
	San Jose Hospital [29, 71]								
	Subtotal/Median/Range	?	O/C	Y		Y		40-43	30-120
	Hospital Infanta Cristina [71]	?	O/C	Y		Y	others	40-43	30-120
	Hospital Santiago Apostol [13]	25	O/C	Y	Y	Y	MMC+CPT-11, 5-FU	40-43	30-90
Subtotal		>25	O/C	Y		Y	MMC+CPT-11, 5-FU	41.5 (40-43)	90 (30-120) /30
Canada, 2	University of Calgary [56, 66, 68, 73]								
	Subtotal/Median/Range	375	O	12-15 mg		400 mg/m^2^		41.5 (40-42)	60
	Maisonneuve-Rosemont Hospital, University of Montreal [14, 38, 53]								
	Subtotal/Median/Range	>40	O	30-50 mg/m^2^	30-50 mg/m^2^ + 50-100 mg/m^2^	360-460 mg/m^2^	L-OHP (360-460 mg/m^2^) + CPT-11 (100-200 mg/m^2^)	41.5 (41-42.5) /43 (42-43)	90 (60-120) /30 or 60
Subtotal		>415	O	12-15 mg /30-50 mg/m^2^	30-50 mg/m^2^ + 50-100 mg/m^2^	360-460 mg/m^2^	L-OHP + CPT-11	41.5 (41-42.5) /43 (42-43)	60 or 90 (60-120) /30 or 60
Greece, 2	Metaxa Cancer Memorial Hospital [29, 71]								
	Subtotal/Median/Range	?	O/C	Y		Y		40-43	30-120
	Didimotichon General Hospital [13]	25	O/C	Y	Y	Y	MMC+CPT-11, 5-FU	40-43	30-90
Subtotal		>25	O/C	Y	Y	Y	MMC+CPT-11, 5-FU	41.5 (40-43)	30-90
Australia, 1	St. George Hospital [15, 26, 29, 34, 41, 45, 64, 72, 81, 90, 91]								
Subtotal		>618	O	10-12.5 mg/m2		350 mg/m^2^		42	90 or 30

Note: C: closed abdomen technique for HIPEC; O: open abdomen technique for HIPEC; Y: yes; MMC: mitomycin C; DDP: cisplatin; 5-FU: fluorouracil; L-OHP: oxaliplatin; CPT-11: irinotecan

**Table 18:** Summary of HIEPC-related procedures in different PC institutions or countries (published researches)

Country /No. Institutions	Major Institutions	No. patients	Mode	HIPEC-MMC alone	HIPEC-MMC+DDP	HIPEC-L-OHP alone	HIPEC-other	Temperature (°C)	Duration (min)
China, 1	Zhongnan Hospital of Wuhan University [39]								
Subtotal		62	O		MMC (30 mg) + DDP (120 mg)			43.0±0.5	90
Norway, 1	Norwegian Radium Hospital [103]								
Subtotal		109	O/C	35 mg/m2				41.4 (39.5-42.1)	90
Denmark, 1	Aarhus University Hospital [60, 75]								
Subtotal		53	O	35 mg/m2				41-42.5	90
Germany, 1	University of Wuerzburg Medical Centre [15, 29, 72]								
Subtotal		>11	O	10-20 mg/m2		Y		42 /40-43	90 /30
Israel. 1	Tel Aviv Medical Center [13]								
Subtotal		25	O/C	Y	Y	Y	MMC+CPT-11, 5-FU	40-43	30-90
Japan, 1	Shizuoka Cancer Centre [13]								
Subtotal		25	O/C	Y	Y	Y	MMC+CPT-11, 5-FU	40-43	30-90
Mexico, 1	Instituto Jalisciense de Cancerologia [29, 72]								
Subtotal		?	O/C	Y		Y		40-43	30-120
Serbia and Montenegro, 1	First Surgical University Hospital, Clinical Center of Serbia [61]								
Subtotal		18	C	10 or 12.5 mg/m2				42	120

Note: C: closed abdomen technique for HIPEC; O: open abdomen technique for HIPEC; Y: yes; MMC: mitomycin C; DDP: cisplatin; 5-FU: fluorouracil; L-OHP: oxaliplatin; CPT-11: irinotecan

**Table 19:** Summary of HIEPC-related procedures in different PC institutions or countries (published researches)

Country /No. Institutions	Major Institutions	No. patients	Mode	HIPEC-MMC alone	HIPEC-MMC+DDP	HIPEC-L-OHP alone	HIPEC-other	Temperature (°C)	Duration (min)
Serbia, 1	Institute for Oncology and Radiology of Serbia [70]								
Subtotal		61	C			410 mg/m2		41	30-60
Singapore, 1	National Cancer Centre Singapore [79, 81]								
Subtotal		63	C	Y				42	60
Sweden, 1	Akademiska Sjukhuset, Uppsala University Hospital [83]								
Subtotal		38	O			460 mg/m2		42-44	30
Total	73	≈6,500	O (n = 63) C (n = 51)	n = 64 30-50 mg/m2 10-12.5 mg/m^2^ 35 mg/m^2^ 10-20 mg/m^2^	n = 24 30-50 mg/m^2^ + 50-100 mg/m^2^	n = 43 460 mg/m2 360-460 mg/m^2^	MMC+CPT-11, 5-FU L-OHP + CPT-11 MMC + CPT-11	41.5 (40-43) /43 (40-43)	90 (60-90) / 60

Note: C: closed abdomen technique for HIPEC; O: open abdomen technique for HIPEC; Y: yes; MMC: mitomycin C; DDP: cisplatin; 5-FU: fluorouracil; L-OHP: oxaliplatin; CPT-11: irinotecan

#### Patients characteristics

In this meta-analysis, the median complete cytoreduction (CC0-1) rate was 72.2% (range, 32.4% - 100%), including 4 studies with 100% CC0 [28, 35, 36, 40], 7 studies with 50% - 99% CC0 [14, 15, 26, 29, 34, 37, 99], and 4 studies with <50% CC0 [12, 13, 38, 39]. Major clinico-pathologic characteristics of the 6,857 CRC PC patients (sample size ranging from 11 to 660) in 61 non-controlled studies are listed by Table 6-10.

#### HIPEC characteristics

Major technical features of HIPEC procedures in each institution are summarized in Table 11-19. HIPEC was performed using only open technique in 22 institutions and only closed techniques 10 institutions, with 41 institutions used both open and closed techniques. The commonly used chemotherapy agents were mitomycin C (MMC) alone (*n* = 63, dosage of 30-50 mg/m^2^ in 88% of institutions, median temperature 41.5°C, ranging from 40 - 43°C, and median duration 90 min, ranging from 60 - 90 min), oxaliplatin (L-OHP) alone (*n* = 43, dosage of 460 mg/m^2^ in 60% of institutions, median temperature 43°C, ranging from 40 - 43°C; and median duration 60 min), and a combination of MMC and cisplatin (CDDP) (*n* = 24, dosage of 30-50 mg/m^2^ + 50-100 mg/m^2^ in 33% of institutions).

### Primary results for meta-analysis

#### Meta-analysis outcomes

**Figure 2:**
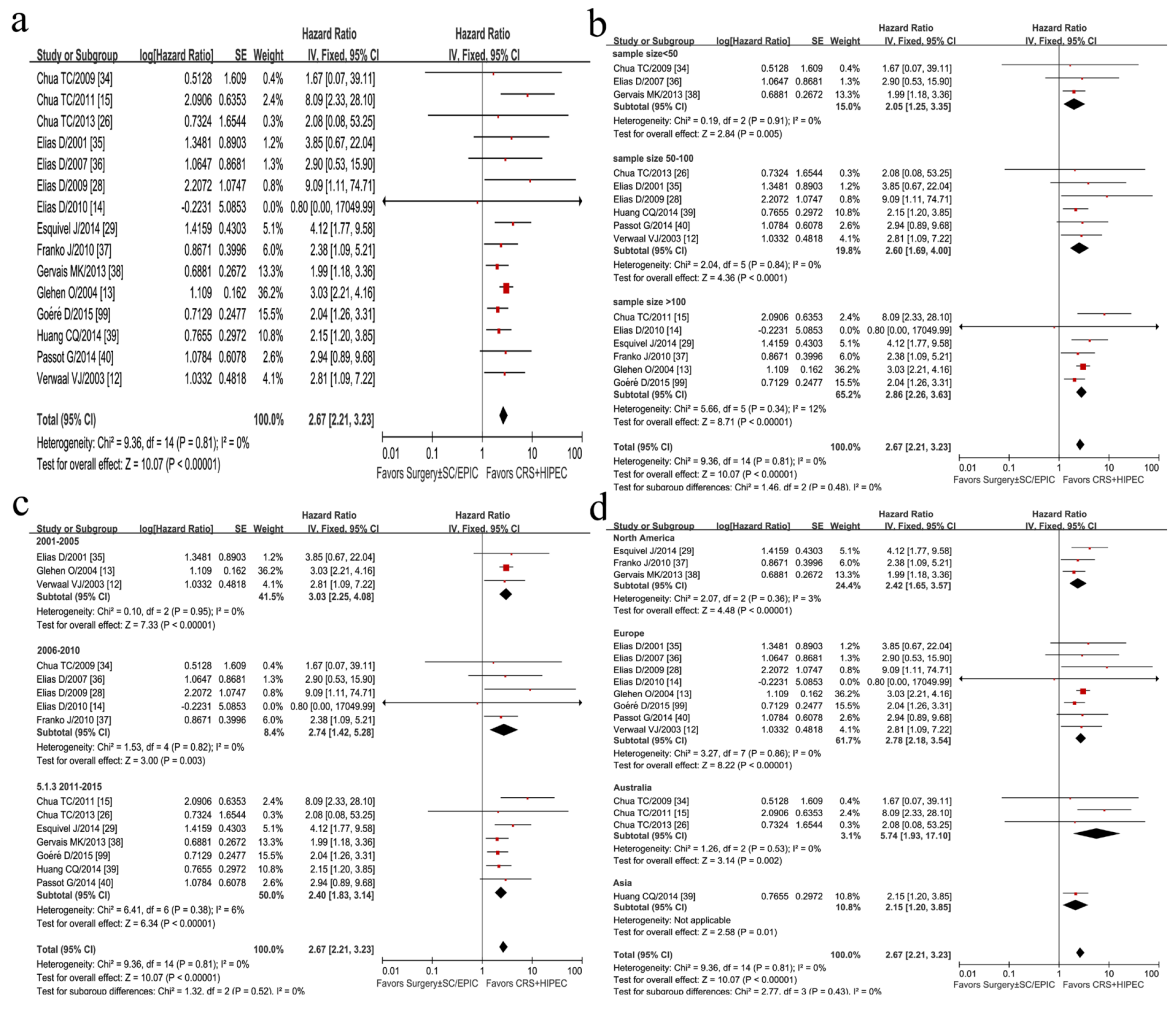
Forest plots of 15 studies displaying the results of the meta-analysis on hazard ratios (HR) for overall survival (OS) (**a**); Sensitivity analysis of sample size difference (**b**), published-time difference (**c**), and geographic-distribution difference (**d**).

The summarized HRs for OS in the 15 controlled researches was 2.67 (95% CI, 2.21-3.23, *I*^2^ = 0%, *P* < 0.00001) (Figure 2a), suggesting that CRC PC patients could obtain more benefits from CRS plus HIPEC than traditional therapy, without apparent heterogeneity among the studies (*P* = 0.81, *I*^2^ = 0%).

Sensitivity analysis of summarized HR and 95% CI showed no difference after choosing random effects model and fixed effects model. In terms of sample size difference, 15 researches were divided into three subgroups (sample size <50, 50-100, >100) by a sensitivity study for a stratified meta-analysis. The summarized HR and 95% CI showed no difference, with no between-subgroup heterogeneity (*P* = 0.48, *I*^2^ = 0%) (Figure 2b). In a sensitivity analysis, four studies with potential heterogeneity was removed due to small sample size [34] or asymmetrical sample size between two groups [14, 26, 40, 99], but the summary HR was 2.81 (95%CI, 2.28-3.48, *I*^2^ = 0%, *P heterogeneity* = 0.56).

There was no statistically significant heterogeneity of HRs for published-time pertinence (*P* = 0.52) (Figure 2c) and geographic-distribution pertinence (*P* = 0.43) (Figure 2d).

#### Analysis of chemotherapy regimens

**Figure 3:**
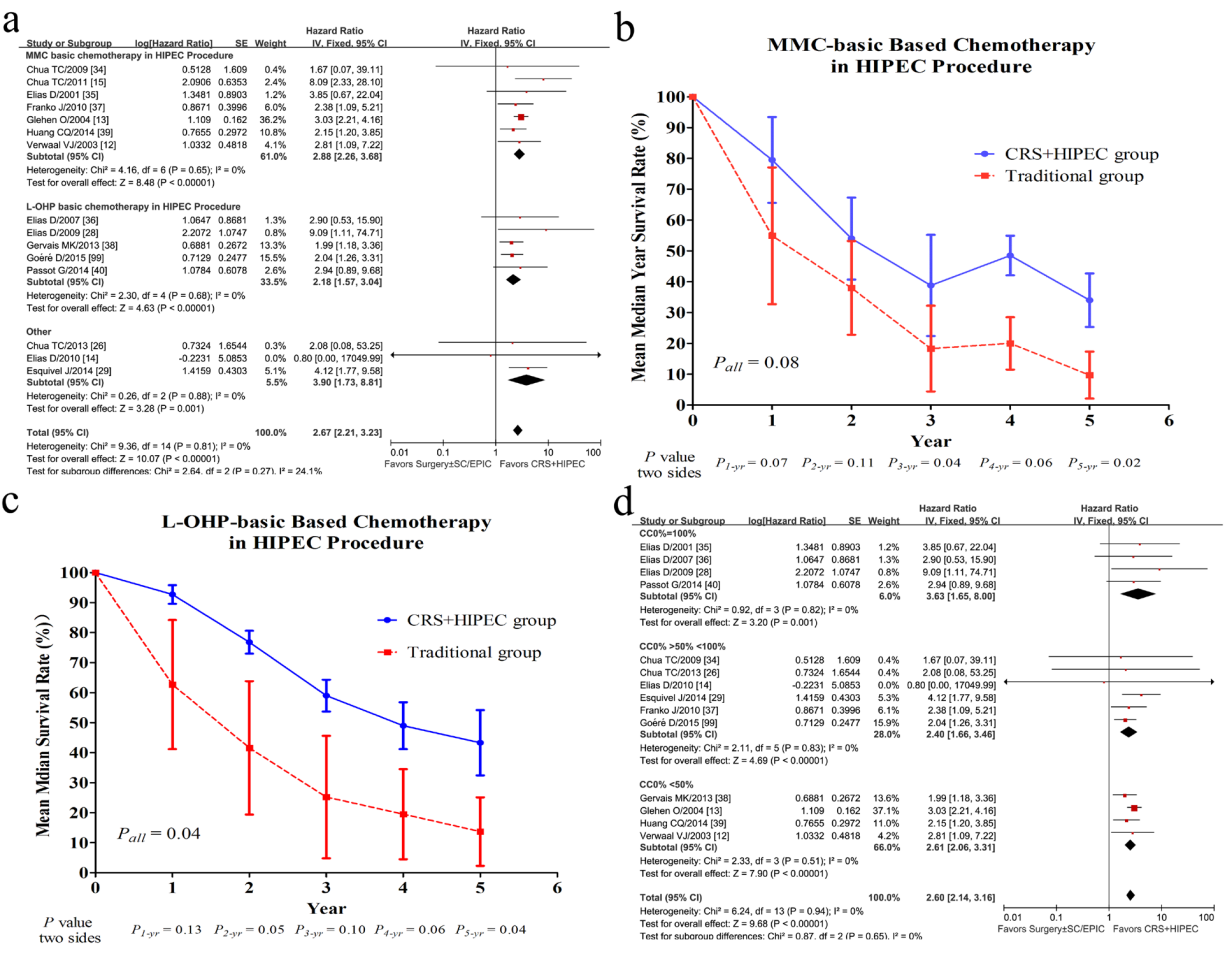
Forest plots of 15 studies evaluating heterogeneity test of chemotherapy regimens difference (MMC based chemotherapy; L-OHP based chemotherapy; others) in HIPEC procedure (**a**); The difference of mean year survival rate between CRS+HIPEC group and traditional group for MMC-basic (Mitomycin C, MMC) HIPEC procedure (**b**), for L-OHP-basic (Oxaliplatin, L-OHP) HIPEC procedure (**c**); Forest plots of 15 studies evaluating heterogeneity test of the proportion of CC0 difference (**d**).

Regarding the effect of different chemotherapy regimens in HIPEC procedure on the efficacy on OS or DFS, 15 researches were divided into 3 subgroups: group of MMC based chemotherapy, group of L-OHP based chemotherapy, and group of other regimens. The heterogeneity showed no significant difference (*P* = 0.27, *I*^2^ = 24.1%), which revealed that difference of chemotherapy regimens of HIPEC was not associated with OS and DFS after CRS and HIPEC in this meta-analysis (Figure 3a). A further analysis of difference in median year survival rate between group of CRS plus HIPEC and group of traditional treatment was conducted by independent-samples *T* test stratified by MMC and L-OHP subgroups (Figure 3b and Figure 3c).

#### MMC-based HIPEC procedure

OS data by MMC-based HIPEC procedure were available in 7 studies with 614 patients [12, 13, 15, 34, 35, 37, 39]. Due to more patients received MMC regimen in studies by Elias et al. [35] (21 patients for MMC regimen, while 6 patients for L-OHP regimen) and Glehen et al. [13] (322 patients for MMC regimen, while 32 patients for L-OHP regimen and 29 patients for others), these two studies were included in MMC subgroup. The stratification analysis showed that OS of patients receiving HIPEC by MMC was significantly improved (HR = 2.88, 95% CI, 2.26-3.68, *I*^2^ = 0%, *P* < 0.00001) (Figure 3a), with 1-, 3-, and 5-year survival rates of 79.5%, 38.8%, and 34%, respectively (Figure 3b). In comparison, the corresponding survival rates in the traditional group were 54.9%, 18.3%, and 9.7%, respectively (Figure 3b).

#### L-OHP-based chemotherapy in HIPEC procedure

Four studies using L-OHP based chemotherapy in HIPEC procedures of 283 patients [28, 36, 38, 40, 99]. A statistically significant benefit for OS was revealed in HIPEC group (HR = 2.18, 95% CI, 1.57-3.04, *I*^2^ = 0%, *P* < 0.00001) (Figure 3a), with the 1-, 3-, and 5-year survival rates of 93%, 59%, and 43%, respectively in HIPEC group *vs*. 63%, 25%, and 14%, respectively in traditional group (Figure 3c).

#### Other chemotherapy regimes in HIPEC procedure

Three trials [14, 26, 29] were identified as the subgroup of other regimen due to difficulties in identifying them as MMC subgroup or L-OHP subgroup since mixed chemotherapy regimens were used in HIPEC during the whole disease course. A significant survival benefit in HIPEC group *vs*. traditional group (HR = 3.90, 95% CI, 1.73-8.81, *I*^2^ = 0%, *P* < 0.00001) was demonstrated (Figure 3a).

#### Publication bias

**Figure 4:**
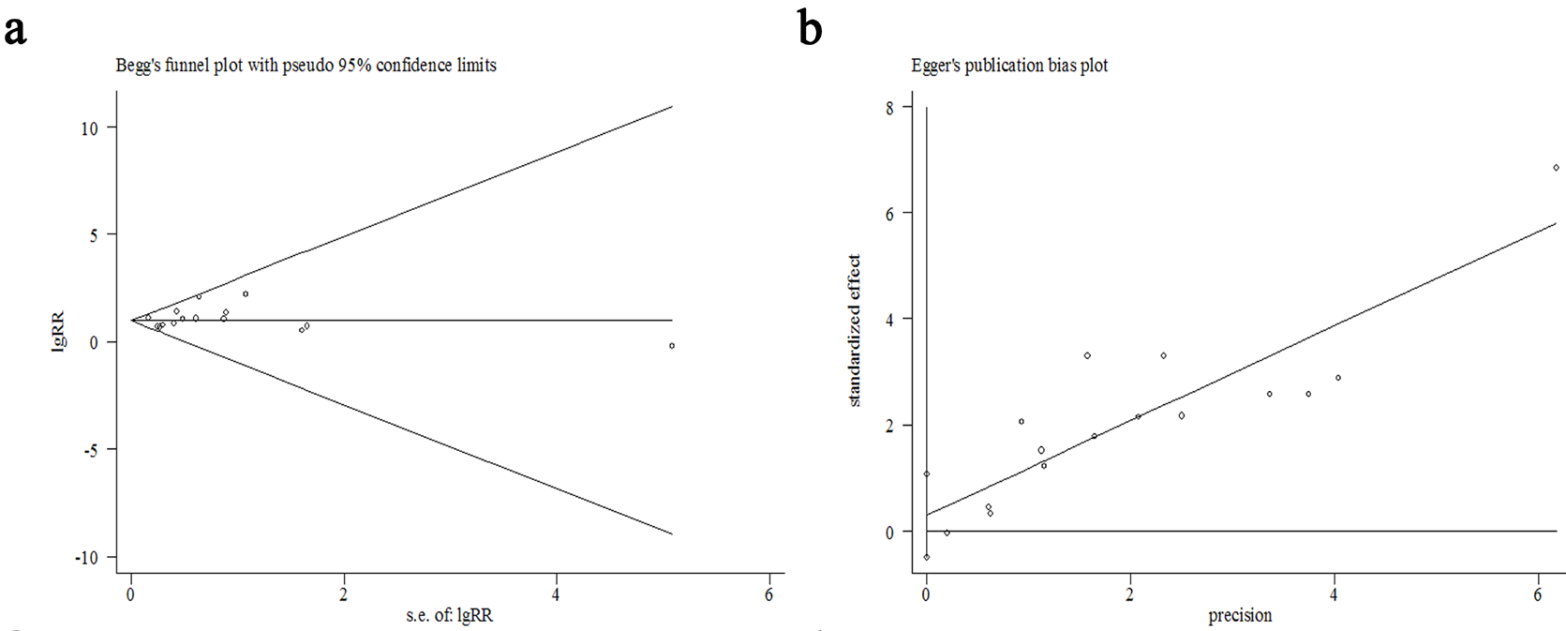
Funnel plots of this meta-analysis by Begg's test (**a**), and by Egger's test (**b**).

Publication bias was evaluated with funnel plot analyses, as shown in Figure 4, and the funnel plot was symmetric. No apparent publication bias was found in our OS meta-analysis with Begg's test (z *continuity corrected* = 0.99, *Pr* >|z|*continuity corrected* = 0.32) (Figure 4a), or with Egger's test (t = 0.82, *P* >|t|= 0.427, 95%CI of bias: -0.49˜1.1) (Figure 4b).

### Summary of HIPEC-related data

**Figure 5:**
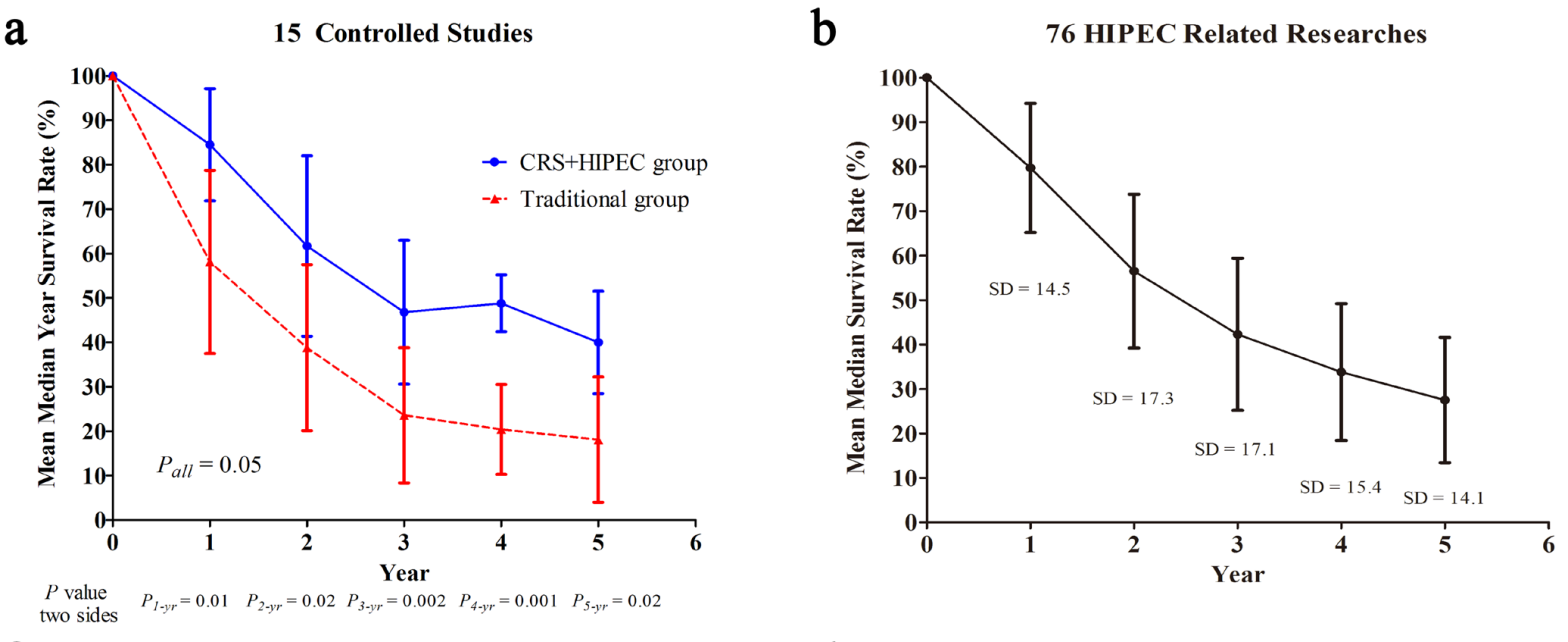
The summarized median year survival rates between CRS+HIPEC group and traditional group for 15 controlled studies (**a**); The summarized median year survival rates on 76 HIPEC related studies (**b**).

In 15 controlled studies and 59 single-arm studies, HIPEC-related outcomes including survival rates, median OS and 95% CI, DFS/RFS, PFS, follow-up time, morbidity, and mortality, are summarized in Table 20-25 and Figure 5

**Table 20:** Survival of Patients with CRC PC Treated by CRS and HIPEC and/or EPIC and/or SC: Summary of 76 Researches

Author/ Years/ Country	1-yr SR (%)	2-yr SR (%)	3-yr SR (%)	4-yr SR (%)	5-yr SR (%)	Mortality Rate (%)	Morbidity Rate (%)	Median OS (mo)	OS 95% CI (mo)	PFS(95% CI) (mo)	DFS/RFS (95% CI) (mo)	Follow-up times (range) (mo)
Controlled Studies												
Chua TC/ 2009/ Australia [34]	≈84	≈50	≈26	NA	NA	NR	NR	13	NR	NR	NR	18 (9-59)
Chua TC/ 2011/ Australia [15]	92	NR	55	NR	30	NR	NR	38	30.2 - 45.2	NR	17 (1-216) (two groups)	17 (1-126)
Chua TC/ 2013/ Australia [26]	NR	NR	NR	NR	41	NR	NR	38	21.1 - 54.9	NR	33 (22.4-43.8) (RFS)	22 (5-88)
Elias D/ 2001/ France [35]	NR	≈70	≈53	≈53	≈44	8.1	Overall: 54.6	≈54	NR	NR	≈26 2-,3-,5-yr 54.7%, 39.4% and 18.4% (two groups)	51.7 (8.1-89.3)
Elias D/ 2007/ France [36]	≈96	≈78	≈63	≈54	54	0	4	NA	NR	NR	NR	113 (70-188)
Elias D/ 2009/ France [28]	NR	81	NR	NR	51	NR	NR	62.7	NR	NR	NR	95.7 *vs*. 63
Elias D/ 2010/ France [14]	NR	NR	40	NR	25.5	NR	NR	31	NR	NR	≈9 1-,3-,5-yr 47%, 15% and 10% (two groups)	NR
Esquivel J/ 2014/ America [29]	NR	NR	66	NR	58	NR	NR	41	38.0-46.3	NR	NR	25 *vs*. 8 (0.1-128)
Franko J/ 2010/ America [37]	≈92	≈66	≈51	≈44	≈28	NR	NR	34.7	NR	NR	NR	NR
Gervais MK/ 2013/ Canada [38]	≈92	≈76	61	≈53	36	4	20	≈54	NR	NR	≈8	22.8 (2-81)
Glehen O/ 2004/ France [13]	NR	NR	NR	NR	NR	NR	NR	21.6/19.2	NR	NR	NR	53 (5-192)
Goéré D/ 2015/ France [99]	≈90	≈72	52	≈40	≈32	5.8	29.5	≈35	NR	NR	NR	60 (47-74)
Huang CQ/ 2014/ China [39]	63.6	20.0	16.0	NR	NR	0	28.6	13.7	10.0-16.5	NR	NR	41.5 (11.5-70.9)
Passot G/ 2014/ France [40]	NR	NR	NR	NR	NR	NR	NR	36	NR	NR	NR	NR
Verwaal VJ/ 2003/ Netherlands [12]	≈66	≈42	≈32	NR	NR	8	19	22.4	NR	NR	NR	21.6
Subtotal of 15 studies (Mean ± SD; Median/Range)	84.5 ± 12.6 *vs*. 58.1 ± 20.6 91 (63.6-96) *vs*. 54 (27.5-87)	61.7 ± 20.3 *vs*. 38.8 ± 18.7 70 (20-81) *vs*. 42 (12-65)	46.8 ± 16.2 *vs*. 23.6 ± 15.2 52 (16-66) *vs*. 18 (0-47)	48.8 ± 6.4 *vs*. 20.4 ± 10.1 53 (44-54) *vs*. 22 (14-33)	40.0 ± 11.5 *vs*. 18.1 ± 14.1 38 (25.5-58) *vs*. 18 (0-44)	4.3 ± 3.7 *vs*. 6.2 ± 4.2 5 (0-8.1) *vs*. 6.3 (0-11.1)	19.8 ± 9.2 *vs*. 20.5 ± 12.3 19.5 (4-29.5) *vs*. 23 (3.1-31.6)	34.3 ± 14.8 *vs*. 18.8 ± 8.8 35 (13-62.7) *vs*. 17 (8.5-34)				43.8 ± 32.8 *vs*. 29.7 ± 29.3 25 (17-113) *vs*. 18 (8-63)

Note: yr: year; SR: survival rate; mo: months; OS: overall survival; PFS: progression-free survival; RFS: recurrence-free survival; DFS: disease-free survival; NA: not achieved; NR: not reported; PMP: pseudomyxoma peritonei; L-OHP: oxaliplatin; MMC: mitomycin; all: all tumors in researches; MVR: multivisceral resection group; NVR: No visceral resection group; APP: appendix; NNT: non-neoadjuvant therapy; NCA: neoadjuvant chemotherapy alone; NCB: neoadjuvant chemotherapy + bevacizumab; AC: adjuvant chemotherapy; NAC: non- adjuvant chemotherapy

**Table 21:** Survival of Patients with CRC PC Treated by CRS and HIPEC and/or EPIC and/or SC: Summary of 76 Researches

Author/ Years/ Country	1-yr SR (%)	2-yr SR (%)	3-yr SR (%)	4-yr SR (%)	5-yr SR (%)	Mortality Rate (%)	Morbidity Rate (%)	Median OS (mo)	OS 95% CI (mo)	PFS(95% CI) (mo)	DFS/RFS (95% CI) (mo)	Follow-up times (range) (mo)
HIPEC single arm studies												
Alzahrani/ 2015/ Australia [41]	≈84	56	≈40	≈26	24	1.2	23.3	28	NR	NR	NR	NR
Beaujard/ 2000/ France [42]	NR	NR	NR	NR	NR	NR	NR	12	NR	NR	NR	NR
Bijelic/ 2008/ Australia [43]	≈94	≈56	≈44	≈23	17	NR	NR	30	NR	15	NR	Mean: 40.8 Median: 29.5
Braam/ 2014/ Australia [44]	NR	NR	NR	NR	6	NR	NR	14.9	NR	NR	11.4	26.2
Cao/ 2009/ Australia [45]	83.6	65.4	51.4	32.1	32.1	NR	NR	37.0	1-72	NR	NR	19 (1-72)
Cavaliere/ 2006/ Italy [46]	NR	NR	25.8	NR	NR	3.3	22.5	19	NR	NR	16	16
Ceelen/ 2014/ Belgium [47]	≈75 (NNT) ≈75 (NCA) ≈96 (NCB)	≈57 (NNT) ≈47 (NCA) ≈89 (NCB)	≈39 (NNT) ≈30 (NCA) ≈71 (NCB)	≈32 (NNT) ≈19 (NCA) NA (NCB)	≈25 (NNT) ≈13 (NCA)	NR	NR	27 (included APP) 24 (Right colon) 27 (Left colon) 35 (Rectal) 25 (NNT) 22 (NCA) 39 (NCB) 30 (AC) 22 (NAC)	20.8-33.2 (included APP) 10.3-37.7 (Right colon) 22.8-31.2 (Left colon) 4.9-65 (Rectum) 19.1-30.9 (NNT) 12.9-31.1 (NCA) 17.6-60.4 (NCB) 20.7-39.3 (AC) 14.2-29.8 (NAC)	NR	NR	18

Note: yr: year; SR: survival rate; mo: months; OS: overall survival; PFS: progression-free survival; RFS: recurrence-free survival; DFS: disease-free survival; NA: not achieved; NR: not reported; PMP: pseudomyxoma peritonei; L-OHP: oxaliplatin; MMC: mitomycin; all: all tumors in researches; MVR: multivisceral resection group; NVR: No visceral resection group; APP: appendix; NNT: non-neoadjuvant therapy; NCA: neoadjuvant chemotherapy alone; NCB: neoadjuvant chemotherapy + bevacizumab; AC: adjuvant chemotherapy; NAC: non- adjuvant chemotherapy

**Table 22:** Survival of Patients with CRC PC Treated by CRS and HIPEC and/or EPIC and/or SC: Summary of 76 Researches

Author/ Years/ Country	1-yr SR (%)	2-yr SR (%)	3-yr SR (%)	4-yr SR (%)	5-yr SR (%)	Mortality Rate (%)	Morbidity Rate (%)	Median OS (mo)	OS 95% CI (mo)	PFS(95% CI) (mo)	DFS/RFS (95% CI) (mo)	Follow-up times (range) (mo)
HIPEC single arm studies												
Desantis/ 2014/ France [48]	≈88	≈72	60.3	≈47	37	1 (all)	12.5 (all)	45.9	NR	NR	16.8 1-,3-,5-yr 61.3%, 30.4% and 22.8%	NR
Elias/ 2004/ France [49]	83	74	65	NR	NR	8.3	41.6	NR	NR	NR	18 1-,2-,3-yr 61%, 50% and 50%	27.4 (18.3-49.6)
Elias/ 2014/ France [50]	91.4	≈74	54	≈47	36.5	4.2	17	≈41	NR	NR	NR	62.4 (55.6-77.6)
Evers/ 2011/ Netherlands [51]	NR	NR	NR	NR	36	NR	NR	49.2 *vs*. 41.3 (Ovarian metastases *vs*. without ovarian metastases)	NR	NR	36.9 *vs*. 32.5 (Ovarian metastases *vs*. without ovarian metastases)	22 (1 week – 97 mo)
Faron / 2016/ France [100]	NR	NR	NR	NR	42	4.6	47	41	32-50	NR	17.7 (12-19) 5-yr: 14%	48.5 (41.0-56.3)
Franko/ 2008/ America [52]	≈79 (MVR) ≈12 (NVR)	≈46 (MVR) ≈30 (NVR)	≈31 (MVR) ≈30 (NVR)	≈16 (MVR) ≈30 (NVR)	0 (MVR) ≈15 (NVR)	1.4	60	20.2 (MVR) 14.3 (NVR)	NR	NR	NR	NR
Frøysnes/ 2016/ Norway [103]	≈93	≈78	65	≈45	36	0	15.1	47	42-52	NR	10 (7-12)	45 (35-55)
Glehen/ 2003/ France [53]	NR	NR	NR	NR	NR	1.8 (all)	28.6 (all)	17.5	4.4-53.6	NR	NR	18.1 (4.4-56) (all)
Glehen/ 2004/ France [16]	55	32	NR	NR	11	4	23	12.8	NR	NR	NR	59.5 (2-148)
Glehen/ 2010/ France [54]	≈80	≈56	41	≈33	26	4.1 (all)	33.6 (all)	30	NR	NR	1-,3-,5-yr 77%, 49% and 37%	45.3 (20.3-90.9) (all)
Gomes / 2005/ America [55]	≈60	≈30	≈20	≈20	0	NR	NR	20	NR	NR	NR	15.7 (1-51)
Gusani/ 2008/ America [56]	≈74	≈49	≈49	≈39	NR	0	29.8 (all)	≈23.6	NR	NR	NR	35.9 (19.0-57.7) (all)
Hamilton/ 2011/ Canada [57]	≈79	≈62	38	≈34	34	NR	NR	27	0-87	NR	9 (0-87) 3-,5-yr 34%,26%	28 (0-119) (all)
Hompes/ 2012/ Belgium [58]	97.9	88.7	≈84	NA	NA	0	52.1	NA	NA	NR	19.8 (12–upper limit not defined) (RFS)	22.7 (3.2-55.7)

Note: yr: year; SR: survival rate; mo: months; OS: overall survival; PFS: progression-free survival; RFS: recurrence-free survival; DFS: disease-free survival; NA: not achieved; NR: not reported; PMP: pseudomyxoma peritonei; L-OHP: oxaliplatin; MMC: mitomycin; all: all tumors in researches; MVR: multivisceral resection group; NVR: No visceral resection group; APP: appendix; NNT: non-neoadjuvant therapy; NCA: neoadjuvant chemotherapy alone; NCB: neoadjuvant chemotherapy + bevacizumab; AC: adjuvant chemotherapy; NAC: non- adjuvant chemotherapy

**Table 23:** Survival of Patients with CRC PC Treated by CRS and HIPEC and/or EPIC and/or SC: Summary of 76 Researches

Author/ Years/ Country	1-yr SR (%)	2-yr SR (%)	3-yr SR (%)	4-yr SR (%)	5-yr SR (%)	Mortality Rate (%)	Morbidity Rate (%)	Median OS (mo)	OS 95% CI (mo)	PFS(95% CI) (mo)	DFS/RFS (95% CI) (mo)	Follow-up times (range) (mo)
HIPEC single arm studies												
Hompes/ 2014/ Belgium [59]	≈91 (L-OHP) ≈88 (MMC)	≈68 (L-OHP) ≈59 (MMC)	≈53 (L-OHP) ≈42 (MMC)	≈45 (L-OHP) ≈33 (MMC)	NA	0	41.1	37.1 (L-OHP) 26.5 (MMC)	22.4-52.8 (L-OHP) 16.9-64.8 (MMC)	NR	12.2 (7.2-undefined) (L-OHP) 13.8(7.0-25.8) (MMC) (RFS)	33.6 (L-OHP) 61.2 (MMC)
Iversen/ 2013/ Denmark [60]	≈97	60	47	38	38	2.9	32.4	≈31	NR	NR	NR	16.0 (0.9–71.3)
Kecmanovic/ 2005/ Serbia and Montenegro [61]	≈85	≈85	≈85	≈85	NA	0	44.4	15	1-57	NR	NR	21 (1-56)
Kianmanesh/ 2007/ France [62]	≈95	72	≈57	44	44	2.3	39	38.4	32.8-43.9	NR	NR	NR
Klaver/ 2011/ Netherlands [63]	71	≈56	≈43	≈35	≈18	NR	NR	28	3-100	NR	NR	NR
Klaver/ 2012/ Netherlands [64]	83	≈52	≈26	≈26	NA	0	33.3	35	20.0-49.9	NR	12 (7.7-16.3)	10.5 (1-52)
Kuijpers/ 2013/ Netherlands [65]	≈84	≈62	46	≈37	31	3 included PMP	34 included PMP	33	28-38	15 (13–17)	NR	41 (35-46) included PMP
Kuijpers/ 2014/ Netherlands [66]	≈87	≈62	45	≈37	≈32	0	30	30	19-41	15 (14-16)	NR	47 (43-51)
Lanuke/ 2009/ Canada [67]	≈85	≈58	≈46	NA	NA	4 (all)	39 (all)	26	1-48	NR	8 (1-31)	12 (1-48)
Levine/ 2014/ America [68]	≈69	≈38	≈27	≈19	≈17	3.8 (all)	42 (all)	≈19	NR	NR	NR	NR
Maillet/ 2016/ France [101]	NR	NR	58	NR	34	4	NR	43.3	NR	12.4	NR	NR
McConnell/ 2013/ Canada [69]	NR	NR	NR	NR	NR	0	36.9	NR	NR	NR	NR	NR
Nikolic/ 2014/ Serbia [70]	78.6	58.7	≈53	≈50	≈42	NR	NR	51	>22	NR	23 (>16) 1-,2-,6-yr 68.3%, 46.7% and 38.1%	22 (1-83)
Passot/ 2012/ France [21]	77	51	NR	NR	33	NR	NR	36.2	NR	NR	NR	58.5 (1-183)
Passot/ 2016/ France [104]	≈83	≈65	≈51	≈38	31	NR	30	36	NR	NR	11	NR
Pilati/ 2003/ Italy [71]	≈68	31	NR	NR	NR	0	35	18	NR	13	NR	14.5
Prada-Villaverde/ 2014/ Spaini [72]	≈85	≈63	≈45	≈38	≈35	NR	NR	31.4	NR	NR	NR	NR
Quenet/ 2011/ France [73]	≈92	≈72	≈36	≈47	≈44	4.1	47.2	41	32–60	NR	15.7 (12–18) (RFS)	48.5 (41.0–56.3)
Rivard/ 2014/ Canada [74]	≈88 (Colon) ≈80 (Rectal)	≈68 (Colon) ≈24 (Rectal)	≈46 (Colon) ≈30 (Rectal)	NA	NA	NR	NR	≈31 (Colon) ≈18 (Rectal)	NR	NR	10.9 3-yr, 15%	30.3 (2-88)

Note: yr: year; SR: survival rate; mo: months; OS: overall survival; PFS: progression-free survival; RFS: recurrence-free survival; DFS: disease-free survival; NA: not achieved; NR: not reported; PMP: pseudomyxoma peritonei; L-OHP: oxaliplatin; MMC: mitomycin; all: all tumors in researches; MVR: multivisceral resection group; NVR: No visceral resection group; APP: appendix; NNT: non-neoadjuvant therapy; NCA: neoadjuvant chemotherapy alone; NCB: neoadjuvant chemotherapy + bevacizumab; AC: adjuvant chemotherapy; NAC: non- adjuvant chemotherapy

**Table 24:** Survival of Patients with CRC PC Treated by CRS and HIPEC and/or EPIC and/or SC: Summary of 76 Researches

Author/ Years/ Country	1-yr SR (%)	2-yr SR (%)	3-yr SR (%)	4-yr SR (%)	5-yr SR (%)	Mortality Rate (%)	Morbidity Rate (%)	Median OS (mo)	OS 95% CI (mo)	PFS(95% CI) (mo)	DFS/RFS (95% CI) (mo)	Follow-up times (range) (mo)
HIPEC single arm studies												
Rodt/ 2013/ Denmark [75]	≈52	≈36	≈12	0	0	0	9.4 (all)	12.7	4.0-21.4	NR	NR	13 (1-44)
Shen/ 2004/ America [20]	NR	NR	25	NR	17	12	30	16	10-26	7 (3-31)	NR	15
Shen/ 2008/ America [76]	91	≈60	48	≈32	26	5.5	41.8	34	23-45	NR	NR	86
Simkens/ 2015/ Netherlands [102]	NR	NR	42	NR	NR	3	24.8	27	18.8-35.3	NR	1-yr: 35%	22.9 (0.4-75.3)
Swellengrebel/ 2009/ Netherlands [77]	NR	NR	NR	NR	NR	NR	NR	25.6	20.9-29.4	13.6 (11.2-16.4)	NR	NR
Tabrizian/ 2014/ America [78]	74.0	≈47	89.4	NA	NA	NR	NR	NR	NR	NR	12.4±1.8 (RFS) 1-,3-yr, 46.9%, 73.9%	15.7±1.2
Teo/ 2013/ Singapore [79]	≈87	≈58	≈36	≈18	≈18	0	56 (all)	≈28	NR	NR	≈10	21 (13.9-31.3) (all)
Teo/ 2014/ Singapore [80]	83.7	≈53	38.2	19.1	19.1	0	40	27.1	15.3-39.1	NR	9.4 (5.5-18.7) 1-,3-,5-yr, 43.8%, 22.3%, 22.3%	24.7 (0.6-81.8)
Ung/ 2013/ Australia [81]	≈84 (Colon)	≈63 (Colon)	≈53 (Colon)	≈37 (Colon)	33 (Colon)	NR	NR	37.1 (Colon) 29.6 (Rectal)	NR	NR	12.6 (Colon) 19.0 (Rectal)	23.3 (1-156) (all)
Vaira/ 2010/ Italy [82]	100 (L-OHP) ≈61 (MMC)	≈60 (L-OHP) ≈17 (MMC)	≈18 (L-OHP) ≈9 (MMC)	≈18 (L-OHP) ≈4 (MMC)	NA (L-OHP) 0 (MMC)	2.5	55	24.6 (L-OHP) 16.6 (MMC)	NR	NR	NR	NR
van Leeuwen / 2008/ Sweden [83]	≈82	≈65	NA	NA	NA	>1 (all)	56.3 (all)	NA	NA	NR	2-yr, 33.5% (all)	13 (2-37) (all)
van Oudheusden/ 2014/ Netherlands [84]	≈86	≈70	≈43	≈30	≈22	1.8	22.1	36.1	NR	NR	NR	16.2 (0.13-90)
van Oudheusden / 2015/ Netherlands [85]	≈87	≈68	44	≈38	≈27	NR	13.5	35.1	NR	NR	NR	12.7(0.10-90.2)
Varban/ 2009/ America [86]	≈63	36.8	≈25	17.4	≈16	7.7	40.1	15.8	13.5-20.2	NR	NR	13.4
Verwaal/ 2005/ Netherlands [19]	75	NR	28	NR	19	NR	NR	21.8	19.0-25.5	NR	NR	46
Votanopoulos/ 2013/ America [87]	≈63 (Colon) ≈83 (Rectal)	≈31 (Colon) ≈36 (Rectal)	25.1 (Colon) 28.2 (Rectal)	NR	NR	5.7 (Colon) 0 (Rectal)	57 (Colon) 46 (Rectal)	17.3 (Colon) 14.6 (Rectal)	NR	NR	NR	88.1 (Colon) 40.1 (Rectal)

Note: yr: year; SR: survival rate; mo: months; OS: overall survival; PFS: progression-free survival; RFS: recurrence-free survival; DFS: disease-free survival; NA: not achieved; NR: not reported; PMP: pseudomyxoma peritonei; L-OHP: oxaliplatin; MMC: mitomycin; all: all tumors in researches; MVR: multivisceral resection group; NVR: No visceral resection group; APP: appendix; NNT: non-neoadjuvant therapy; NCA: neoadjuvant chemotherapy alone; NCB: neoadjuvant chemotherapy + bevacizumab; AC: adjuvant chemotherapy; NAC: non- adjuvant chemotherapy

**Table 25:** Survival of Patients with CRC PC Treated by CRS and HIPEC and/or EPIC and/or SC: Summary of 76 Researches

Author/ Years/ Country	1-yr SR (%)	2-yr SR (%)	3-yr SR (%)	4-yr SR (%)	5-yr SR (%)	Mortality Rate (%)	Morbidity Rate (%)	Median OS (mo)	OS 95% CI (mo)	PFS(95% CI) (mo)	DFS/RFS (95% CI) (mo)	Follow-up times (range) (mo)
HIPEC single arm studies												
Winer/ 2014/ America [88]	53	22	22	≈13	≈13	6.7	22.2	12.2	7.5-17.2	9.3 (3.3-17.8) 1-,3-yr, 47%, 16%	NR	52.8 (12.5-138)
Witkamp/ 2001/ Netherlands [89]	NR	45	23	NR	NR	3	38	NR	NR	NR	11 (3-29) (RFS)	38 (26-52)
Yan/ 2006/ Australia [90]	72	64	NR	NR	NR	0	NR	29	2-39	NR	NR	12 (2-39)
Yan/ 2008/ Australia [91]	79	67	39	NR	NR	NR	NR	29	1-56	NR	NR	14 (1-56)
Zanon/ 2006/ Italy [92]	≈75	≈60	≈28	NR	NR	4	24	30.3	17.0-52.2	17.3 (5.72-28.9)	NR	NR
Total of 76 studies (Mean±SD; Median/Range)	79.7 ± 14.5; 83 (12-100)	56.5 ± 17.3; 60 (17-89)	42.3 ± 17.1; 42 (9-89.4)	33.8 ± 15.4; 34.5 (0-85)	27.5 ± 14.1; 31 (0-58)	2.8 ± 2.9 2.5 (0-12)	33.0 ± 13.4 32.9 (4-60)	29.2 ± 11.3 29 (12-62.7)		13.1 ± 3.2 13.6 (7-17.3)	15.9 ± 7.7 12.6 (8-36.9)	33.1 ± 22.5

Note: yr: year; SR: survival rate; mo: months; OS: overall survival; PFS: progression-free survival; RFS: recurrence-free survival; DFS: disease-free survival; NA: not achieved; NR: not reported; PMP: pseudomyxoma peritonei; L-OHP: oxaliplatin; MMC: mitomycin; all: all tumors in researches; MVR: multivisceral resection group; NVR: No visceral resection group; APP: appendix; NNT: non-neoadjuvant therapy; NCA: neoadjuvant chemotherapy alone; NCB: neoadjuvant chemotherapy + bevacizumab; AC: adjuvant chemotherapy; NAC: non- adjuvant chemotherapy

### Adverse events

In controlled studies, the mean (± SD) mortality and morbidity rates were 4.3% (± 3.7%) and 19.8% (± 9.2%) in HIPEC groups, 6.2% (± 4.2%) and 20.5% (± 12.3%) in traditional groups, respectively (Table 20-25). No significant difference in mortality (*P* = 0.423) or morbidity (*P* = 0.815) was detected between HIPEC group and traditional group by *T* test. In the integrated HIPEC-related data of 76 studies, mean mortality and morbidity was 2.8% (± 2.9%) and 33.0 (± 13.4%), respectively.

## DISCUSSION

Due to the tumor biologic characteristics of colorectal cancer, about 10-13% patients have already progressed to PC when CRC is diagnosed [3, 7], which has a poor prognosis. In order to improve the efficacy, a comprehensive treatment strategy with combination of CRS plus HIPEC had been developed. With wide application of this treatment, CRS plus HIPEC has been proved capable to achieve better survival in selected patients with PC from colorectal cancer.

This meta-analysis of 15 controlled studies demonstrated that CRS+HIPEC comprehensive therapeutic strategy could bring significant survival benefit for selected CRC PC patients than traditional treatment of palliative surgery alone or systemic chemotherapy (HR = 2.67, 95% CI 2.21-3.23, *P* < 0.00001). In addition, the summarizing analysis of these 76 studies showed that the median OS was about 29 months in HIPEC group, which is significant longer compared with median OS of 17.9 months for CRC PC patients receiving contemporary chemotherapy reported by Kerscher et al (*n* = 2,406) [7]. These results provide further supporting evidence that CRS+HIPEC as the principal comprehensive treatment can bring more survival benefit to selected patients with CRC PC than traditional therapy.

The different regimens used in chemotherapy may be one potential confounding factor for survival outcomes. In order to investigate the influence of chemotherapy regimens on postoperative survival, a stratification analysis between MMC based regimens and L-OHP based regimens was conducted. The results of heterogeneity showed no significant difference (*P* = 0.50). These results are inconsistent with the reports by Elias et al [14], which showed that OS advantage for L-OHP regimens over non-L-OHP regimens (32 *vs*. 25 months, *P* = 0.02). However, L-OHP used in HIPEC was not an independent prognostic factor for survival in the study of Elias and colleagues. A multi-center retrospective controlled study reported by Prada-Villaverde et al. [72] showed that of 539 patients undergoing CRS plus HIPEC, L-OHP based HIPEC and MMC based HIPEC achieved similar median OS (31.4 *vs*. 32.7 months, *P* = 0.925). Similarly, the study of Hompes et al. [59] yielded the same conclusion that there was not obvious benefit in OS for HIPEC with L-OHP (37.1 months) or MMC (26.5 months) (*P* = 0.45). Although different chemotherapy regimens in HIPEC may have an effect on stability and reliability of this meta-analysis, the result of heterogeneity analysis was in accordance with above studies. As a result, both MMC and L-OHP were the feasible chemotherapy drugs in HIPEC for CRC PC patients to achieve similar efficacy.

Moreover, there are also some doubts that different chemotherapy in intravenous or postoperative intraperitoneal therapy regimens, even targeted therapy, had interference on the survival outcomes in meta-analysis. The doubts were removed by the report of Kerscher et al [7]. In 2,406 CRC patients of no-PC and PC, the survival outcomes for contemporary chemotherapy regimens (oxaliplatin or irinotecan) were compared with 5-FU regimens. For the CRC patients (without PC), survival outcomes for contemporary regimens were increased over 5-FU regimens (5-year survival rate 71.6% *vs*. 63.3%, *P* = 0.001). On the contrary, for patients with PC from CRC, the survival of L-OHP or irinotecan agent was similar to 5-FU regimens (*P* > 0.05), regardless of synchronous PC (2-year survival rate 31.1% *vs*. 19.1%, *P* = 0.092, and 5-year survival rate 20.8% *vs.* 5.8%, *P* = 0.081) or metachronous PC (2-year survival rate 71.5% *vs*. 58.5%, *P* = 0.329, and 5-year survival rate 28.1% *vs*. 24.4%, *P* = 0.411).

There were a few statistical flaws in this meta-analysis. For example, only one RCT [12] was included. It may be due to the difficulty of performing RCT. Therefore, we had to select meticulously current studies of best evidence level besides the only RCT. However, this meta-analysis showed acceptable outcomes of low heterogeneity and sensitivity. Regrettably, a patient-level (based on single patient data) meta-analysis as the gold standard for meta-analysis was not performed because of the difficulty in obtaining vast data from each database or institution. In addition to meta-analysis, this report provided a summary of 76 clinical studies published until today about CRS and HIPEC, which can get a review of published studies. In order to get the best evidence level results, more RCTs and prospective, multicenter, large-scale clinical trials need to be performed in future studies.

Observing available data from 6 controlled studies (a total of 470patients) [12, 35, 36, 38, 39, 99], mortality or morbidity were found similar in both groups of HIPEC and traditional surgery, which was 4.3% *vs*. 5.0% and 19.8% *vs*. 19.5%, respectively. The summarized HIPEC-related mortality and morbidity in 48 articles (the total number of patients, *n* = 4,809) [12, 16, 20, 35, 36, 38, 39, 46, 48-50, 52-54, 56, 58-62, 64-69, 71, 73, 75, 76, 79, 80, 82-90, 92, 100-104] were 2.8% (SD, ± 2.9%; range, 0-12%) and 33.0% (SD, ± 13.4%; range, 4-60%), respectively. Some large-sample retrospective studies and population-based analysis found a series of approximate results that the range of mortality was 2%-5.6% and morbidity was 25%-34% [93-97]. Furthermore, a systematic review of morbidity and mortality for CRS+HIPEC by Chua et al. [98] showed that the mortality and morbidity range from 0.9% to 5.8% and 12% to 52%, respectively. Though evidence proved that safety for CRS+HIPEC was acceptable, a meta-analysis on mortality and mortality for CRS+HIPEC may be able to provide more convincing results on the mortality and morbidity.

With the summary of 76 studies, it is found that although HIPEC is now widely accepted and performed in most institutions, details of performing HIPEC varies among different institutions. As we noted, there are several mainly different techniques concerning HIPEC including 1) “open” or “closed” technique, 2) using MMC and/or L-OHP, 3) mono-chemotherapy or combination of chemotherapy regimens, 4) temperature and duration of HIPEC. These can be further studied in future studies.

In conclusion, this meta-analysis showed that CRS+HIPEC comprehensive therapeutic strategy was associated with improvement of OS in CRC PC patients, and the results of the meta-analysis were proved of good reliability by low heterogeneous and robust sensitivity. Meanwhile, CRS and HIPEC can be performed with acceptable safety according to summary results of all 76 studies.

## MATERIALS AND METHODS

### Search strategy

The following databases were systematically searched up to July 31, 2016 including PubMed, Science Citation Index, EMBASE, and MEDLINE. The Cochrane Central Register of Controlled Trials, the National Institutes of Health trial registry, and conference proceeding articles from major oncologic and gastrointestinal cancer meetings were also sought for published results. The key words included “colon”, “rectum”, “colorectal”, “cancer”, “peritoneal carcinomatosis”, “hyperthermic intraperitoneal chemotherapy”, and synonyms and related terms for these words. The MeSH terms included “colon cancer”, “rectal cancer”, “colorectal cancer”, “peritoneal carcinomatosis”, “hyperthermic chemotherapy”, “hyperthermic intraperitoneal chemotherapy”, “HIPEC”, “intraperitoneal chemohyperthermia”, and “IPCH”. The combined application of “key words terms” and “MeSH terms” were conducted to improve the efficiency and accuracy of literature search.

### Selection criteria

For inclusion in the meta-analysis and summarized HIPEC-related data analysis, a study had to fulfill the following criteria: (1) According to the North-England evidence-based guidelines [105, 106], excluded from IV levels evidence of literatures were included; (2) All patients were diagnosed CRC PC; (3) For assessing CRS+HIPEC±SC/EPIC, the intervening measure group was CRS+HIPEC±SC/EPIC, while the control group was traditional therapy of surgery and/or SC; For systematic review of CRS+HIPEC to treat CRC PC, HIPEC-related literatures involving clinical efficacy evaluation were included; (4) The key outcome measures should be included in literatures, such as OS, disease-free survival (DFS), recurrence-free survival (RFS), progression-free survival (PFS), year survival rate, morbidity and mortality [107], multivariate analysis, follow-up times; (5) English language; (6) To reduce the effect of publication bias, both fully published articles and abstracts were eligible for inclusion.

Exclusion criteria: (1) Animal studies, pathological research, imageology research, pharmacokinetics research, quality of life assessment, literature review, commentary, letter, book, etc; (2) Duplicate publication or overlapping data (chose the largest and latest sample size); (3) The sample size is less than 10; (4) Multiple cancer; (5) Unresectable liver metastases or others distant metastasis; (6) missing rate of follow-up > 5%.

### Data extraction

Three authors analyzed data from a meta-analysis of 15 controlled researches of CRS plus HIPEC group *vs*. surgery and/or SC group and a summarized analysis of 76 researches of HIPEC group. The following data were extracted from each article: (1) Major clinico-pathologic characteristics and detail HIPEC regimens; (2) Survival and advent events. All relevant text, tables, and figures were reviewed for data extraction. For equivocal literatures or discrepancies between two independently assessed reviewers, these were resolved by discussion and consensus with a third author.

### Statistical methods

All meta-analysis were performed using Review Manager 5. Overall survival (OS) or disease-free survival (DFS) in all studies were extracted from original literature. If not achieved accurate data in original text, hazard ratios (HRs) for time-to-event outcomes with 95% confidence intervals (95% CI) in two groups were estimated by Tierney's methods [108]. The heterogeneity in the meta-analysis was evaluated by *I*^2^ statistics [109] and *T* test [110] was calculated for each result in summarizing analysis of all HIPEC-related data from the included 76 articles. If *I*^2^ >50%, it was defined as the unacceptable heterogeneity. If *I*^2^ <50%, fixed effect model was used to get pooled HR and 95% CI; otherwise, random effects model was used if moderate heterogeneity. For a sensitivity analysis, we investigated the different research features of eligible trials, which included statistical methods, methodological quality, sample sizes, and clinical factors on HIPEC-related effect, after that, summarizing each subgroup data in term of Mental-Haenszel stratification analysis. According to Egger's test [111] and Begg's test [112], publication bias was considered to be inevitable when *P* < 0.10. The funnel plot analyses using ‘STATA: Data Analysis and Statistical Software version 12.0’, was to observe the results of meta-analysis whether any publication bias.
